# Identification and Evaluation of Single-Nucleotide Polymorphisms in Allotetraploid Peanut (*Arachis hypogaea* L.) Based on Amplicon Sequencing Combined with High Resolution Melting (HRM) Analysis

**DOI:** 10.3389/fpls.2015.01068

**Published:** 2015-12-02

**Authors:** Yanbin Hong, Manish K. Pandey, Ying Liu, Xiaoping Chen, Hong Liu, Rajeev K. Varshney, Xuanqiang Liang, Shangzhi Huang

**Affiliations:** ^1^Peanut Research Center, Crops Research Institute, Guangdong Academy of Agricultural Sciences, Guangzhou, China; ^2^School of Life Sciences, Sun Yat-Sen UniversityGuangzhou, China; ^3^Center of Excellence in Genomics, International Crops Research Institute for the Semi-Arid TropicsHyderabad, India; ^4^College of Agriculture, South China Agricultural UniversityGuangzhou, China; ^5^School of Plant Biology and Institute of Agriculture, The University of Western AustraliaCrawley, WA, Australia

**Keywords:** SNPs, peanut, tetraploid, high resolution melting (HRM), polymorphism

## Abstract

The cultivated peanut (*Arachis hypogaea* L.) is an allotetraploid (AABB) species derived from the A-genome (*Arachis duranensis)* and B-genome (*Arachis ipaensis*) progenitors. Presence of two versions of a DNA sequence based on the two progenitor genomes poses a serious technical and analytical problem during single nucleotide polymorphism (SNP) marker identification and analysis. In this context, we have analyzed 200 amplicons derived from expressed sequence tags (ESTs) and genome survey sequences (GSS) to identify SNPs in a panel of genotypes consisting of 12 cultivated peanut varieties and two diploid progenitors representing the ancestral genomes. A total of 18 EST-SNPs and 44 genomic-SNPs were identified in 12 peanut varieties by aligning the sequence of *A. hypogaea* with diploid progenitors. The average frequency of sequence polymorphism was higher for genomic-SNPs than the EST-SNPs with one genomic-SNP every 1011 bp as compared to one EST-SNP every 2557 bp. In order to estimate the potential and further applicability of these identified SNPs, 96 peanut varieties were genotyped using high resolution melting (HRM) method. Polymorphism information content (PIC) values for EST-SNPs ranged between 0.021 and 0.413 with a mean of 0.172 in the set of peanut varieties, while genomic-SNPs ranged between 0.080 and 0.478 with a mean of 0.249. Total 33 SNPs were used for polymorphism detection among the parents and 10 selected lines from mapping population Y13Zh (Zhenzhuhei × Yueyou13). Of the total 33 SNPs, nine SNPs showed polymorphism in the mapping population Y13Zh, and seven SNPs were successfully mapped into five linkage groups. Our results showed that SNPs can be identified in allotetraploid peanut with high accuracy through amplicon sequencing and HRM assay. The identified SNPs were very informative and can be used for different genetic and breeding applications in peanut.

## Introduction

Among all the structural variations in the genomes of animals and plants, single nucleotide polymorphisms (SNPs) provide the most frequent polymorphism having potential to be used as genetic markers. The SNP markers have proven their usefulness in conducting different genetic and breeding studies in a large number of organisms including several crop plants. The SNPs are now preferred by geneticists and breeders in conducting large scale genome-wide association studies (GWAS) and modern breeding such as marker-assisted backcrossing (MABC), marker-assisted recurrent selection (MARS), and genomic selection (GS). To a large extent, these studies have been driven by technology which made available a number of cost-efficient and robust high throughput SNP genotyping platforms. Initially all the analytical softwares for highthrouput genotyping data were designed keeping diploid human genome in mind. Lack of appropriate analytical tools/softwares for polyploidy was the main bottleneck which restricted their use in only major diploid crop plants. Nevertheless, diploid crop plant species benefitted hugely by these new genotyping platforms (Ganal et al., 2009) while polyploids could not avail the benefits of these technologies for many years.

In the allopolyploid crop, the homologous SNPs arise between identical chromosome pairs of either each subgenome, or the corresponding diploid progenitors. Further, the homoeologous sequence variants (HSVs) arise between corresponding nucleotide coordinates of subgenomes (Somers et al., 2003) while the paralogous sequence variants (PSVs) arise between duplicated genes of a genome which diverged from a common ancestor (Fredman et al., 2004). In case of genetic and breeding applications in allopolypoid taxa, only SNPs are useful and not the HSVs or PSVs. SNP identification requires sequencing of different accessions or varieties and the appropriate analytical tools to unambiguously discriminate polymorphic SNPs between homologous and homeologous chromosomes. Unless genome-specific markers are being used, essentially all the SNP marker analysis systems have to deal with the sampling of both the copies of the two homeologous genomes in most cases (Parkin et al., 1995). In this case, instead of showing the usual segregation at a single locus in diploid organisms (AA, AB, and BB), an allotetraploid can segregate in up to five groups (AAAA, AAAB, AABB, ABBB, and BBBB) if the polymorphism is present in both the genomes. In case, the SNPs in specific genotype of allotetraploid were coincident with HSV in another genotype, structures such as CT[A genome]TT[B genome] × CC[A genome]TT[B genome] fail to generate allelic segregation patterns in bi-parental crosses.

In recent years, the next-generation sequencing (NGS) technologies together with improved and customized analytical tools for allotetraploids facilitated successful discovery of large scale SNPs in allopolyploid taxa (Kaur et al., 2012). However, despite using sophisticated bioinformatics tools, homologous sequences obtained by NGS cannot be totally discriminated from homoeologous and paralogous sequences. It will cause the problem that a part of HSVs and PSVs to be misidentified as SNPs. It has been observed that the SNP identification using NGS in allopolyploid without using reference genome sequence has a high false discovery rate of >20% (Kaur et al., 2012). Although, the amplicon sequencing showed lower efficiency than NGS in SNPs identification but the identified SNPs had good quality and reliability (Ganal et al., 2009). The amplicon sequencing approach has not only identified high quality and reliable SNPs, HSVs and PSVs, but also succeeded in anchoring SNP into subgenome in obligate outbreeding allotetraploid forage legume white clover (2*n* = 4x = 32; Hand et al., 2008). The other important advantage of amplicon is possibility to identify haplotypes because of sequencing of larger fragments. This approach is also resource and cost-saving as the pooled DNA samples from many lines can be analyzed in a single sequencing reaction to identify allele frequencies for identified SNPs.

The high resolution melting (HRM) curve analysis is a highly sensitive and efficient method for mutation theory and SNP genotyping (Cho et al., 2008). The advantage of this approach is detection of SNPs without generating sequencing data on the genotypes, therefore, widely been utilized in clinical, vertinery, and cancer research. This approach compare resultant melt profiles to identify homozygous and heterozygous sequence variants on the basis of variations in the shape and position of the melt curve (Croxford et al., 2008). The HRM analysis uses new generation of fluorescent dsDNA dyes which has low toxicity to PCR which allows its use at high concentration to saturate the dsDNA PCR product (Wittwer et al., 2003). The above mentioned feature enhances greater dye saturation leading to less dynamic dye redistribution in non-denatured regions of nucleic strands to provide higher fidelity to fluorescent signals (Wittwer et al., 2003; Monis et al., 2005). Because of the above facts, this method provides greater melting sensitivity and higher resolution melting profiles which allows to detect SNPs in PCR amplicons even in somatic mutations and methylations (Kristensen et al., 2008; Vossen et al., 2009; Chang et al., 2014; Mastoraki et al., 2015). Although the HRM has been successfully deployed for mutation detection and SNP genotyping in medical research (Erali et al., 2008) while its application in plants is still very limited.

Cultivated peanut (*Arachis hypogaea* L.), a self-pollinating allotetraploid (AABB, 2*n* = 4x = 40), is a major oilseed and food crop in semi-arid tropic regions of the world and serve as a significant source of oil and protein to the consumers. The cultivated peanut is derived from a fusion of the *Arachis duranensis* (A genome, *n* = 10) and the *Arachis ipaensis* (B genome, *n* = 10) ~3500 years ago (Hammons, 1994; Seijo et al., 2004). This species has a narrow genetic base and therefore, very low DNA polymorphism making genomic resources development and deployment a formidable task (Pandey et al., 2012; Varshney et al., 2013). So far most of the genetic and breeding studies in peanut have been conducted using SSR markers whose numbers are not enough to conduct comprehensive genetic studies. Nevertheless, few efforts reported development of SNPs and construction of genetic maps (Nagy et al., 2012; Bertioli et al., 2014; Zhou et al., 2014), but these numbers are not adequate. Recently a study reported use of next-generation double-digest restriction-site-associated DNA sequencing (ddRADseq) technique to construct reduced representation libraries (RRLs) for two *A. hypogaea* lines and their recombinant inbred lines (RILs) (Zhou et al., 2014). Although this study detected 53,257 SNPs between the parents, only 14,663 SNPs could be detected in the population, and finally only 1765 high quality polymorphic SNPs were used for genetic map construction. Lack of reference genome sequence and parameters of sequence variation are the obvious reason behind the low SNP validation rate. Although SNPs developed by NGS are cost-effective, but with the low validation rate of SNPs the cost and difficulty of SNPs genotyping will increase substantially in the later stage. Therefore, it will be more appropriate to check initially the frequency of SNPs, HSVs and PSVs in peanut genome, and accordingly the most suitable strategy need to be adopted for developing large scale SNPs. The present study was planned to address some of the above issues with the objectives (1) to identify EST-SNPs and genomic-SNPs in peanut by using amplicon sequencing, (2) to estimate SNPs, HSVs and PSVs frequencies in the peanut genome, (3) to assess the distribution of SNPs in South China peanut varieties, and (4) to map SNPs markers into linkage groups of peanut by using HRM curve analysis.

## Materials and methods

### Plant materials and DNA isolation

A total of 14 genotypes, sequencing test panel, were used for SNP identification. This sequencing panel included 12 tetraploid (AABB) peanut varieties (Addition File 1) and two diploid progenitor's i.e., *A. duranensis* (AA) and *A. ipaensis* (BB). All these genotypes were accessed from Oil Crops Research Institute (OCRI), Chinese Academy of Agricultural Sciences (CAAS), Wuhan, China. Ninety six peanut varieties in south China including the above 12 varieties were investigated for allele frequency (Addition File 1). In addition, the RIL population Y13Zh (Zhenzhuhei × Yueyou13) described by Hong et al. (2010) was used for SNPs validation and subsequent genetic linkage mapping. Total genomic DNA was extracted from young leaves of *A. hypogaea* and its diploid progenitors according the protocol used by Moretzsohn et al. (2005).

### PCR amplification

The expressed sequence tags (ESTs) from peanut EST database developed by Guo et al. (2008) and genome survey sequences (GSS) of *A. hypogaea* available at the NCBI database (www.ncbi.nlm.nih.gov) were accessed for analysis. The primer pairs were designed using these sequences with the Primer Premier 5 program (Whitehead Institute for Biomedical Research, Cambridge, USA). The main parameters for designing primers include (1) 55–65°C melting temperature (Tm) with 60°C as optimum; (2) 450–750 bp product size; (3) 18–24 bp primer length with predicted amplification rate >80%; and (4) 40–60% GC content. The PCR amplification was carried out in a 50 μl volume reaction containing 2 μl of DNA (~100 ng), 1 × Power Pfu buffer (Bioteke, Bejing, China), 1 × enhancer solution (Invitrogen, Carlsbad, CA, USA), 0.2 mM dNTPs, 0.25 μM primers, and 1.25 U Power Pfu DNA Polymerase (Bioteke, Bejing, China). The PCR profile had 1 cycle of 5 min at 94°C, an annealing temperature of 55°C for 35 cycles (1 min at 94°C, 30 s at 55°C, 45 s at 72°C) and an additional cycle of 10 min at 72°C. The presence of a single defined fragment in the test panel was visualized in agarose gel electrophoresis.

### Sanger sequencing and SNP discovery

The PCR products with a single fragment from the 12 peanut varieties and putative donors of subgenomes were purified using the Bioteke PCR Purification Kit (Bioteke, Beijing, China) and sent to BGI (Shenzhen, China) for sequencing. Each PCR product was sequenced from both sides using the Big Dye terminator system, version 3.1 Mix (Applied Biosystems) by an ABI 3730XL DNA sequencer. Sequence alignments and SNP identification were carried out using Mutation Surveyor® (Minton et al., 2011).

### SNPs genotyping

SNPs genotyping in 96 peanut varieties was performed using HRM method. Based on the sequences of amplicons, nested PCR primers were design for HRM analysis at the flanking sequence of SNPs. Primer pairs were designed based on the following core criteria: (1) 80–250 bp of product size; (2) only one SNP from each amplified fragment; (3) primer binding region containing HSV and PSV for designing subgenome specific primer. PCR was performed in a 10 μl reaction under the same conditions as mentioned above, except two modifications. The first modification was addition of 2.5 μM CYTO®9 (Invitrogen, Carlsbad, USA) to the reactions while using either the 1 μl of a 100X dilution of the first PCR (unpurified) or 1 μl of 20X dilution of purified first PCR product as the template. The PCR and HRM analysis were carried out using the LightCycler® 96 Real-Time PCR System (Roche, Basel, Switzerland). The conditions used had 1 cycle at 95°C for 3 min; 40 cycles at 95°C for 10 s, 60°C for 15 s, 72°C for 10 s; 1 cycle at 72°C for 90 s followed by melting at 72°C to 90°C with increase of 0.1°C and waiting time of 2 s each step. After monitoring the amplification status, the significantly early and late amplifications were not considered for HRM analysis to avoid rising of aberrant melting curves. The selected samples were checked on 2% agarose gels for their specificity.

### Statistical analysis

The formula suggested by Botstein et al. (1980) and Anderson et al. (1993) were used for estimation of polymorphism information content (PIC) values for all the SNPs:

$$\text{PIC}=1-\sum_{i\;=\;1}^{k}p_{i}^{2}$$

where *k* is the total number of alleles and p is the frequency of the *i*th allele at a given locus.

### Genetic mapping

Three RIL (recombinant inbred line) populations namely Y13ZH, Y13FU, and Y13J11 were developed from three crosses for conducting genetic mapping (Hong et al., 2010). Among these three RILs, the population Y13ZH displayed highest polymorphism (Hong et al., 2010) and was, therefore, used for SNP mapping in this study. Yueyou 13 (Y13), a Spanish type with high yield, was used as the female parent while Zhenzhuhei, a Virginia type with dark purple testa and high protein (32.4%) content, was used as male parent in developing the population Y13ZH. The population consisted of 142 individual lines. Parental genotypes and 10 selected lines from the RIL mapping population Y13Zh were screened to test the Mendelian segregation of putative SNPs. Validated SNPs were then used for genotyping the entire RIL population in order to integrate SNP markers into the existing peanut genetic map using Joinmap 3.0 as described previously (Hong et al., 2010).

## Results

### Identification of SNPs, HSVs, and PSVs

Amplicon sequences derived from ESTs and GSS were analyzed to identify SNPs in a panel of 12 peanut varieties and two putative diploid progenitors of *A. hypogaea*. Approximately 90% ESTs and GSS showed successful amplification and yielded PCR products. As the ESTs were not screened for the absence of introns at the DNA level, some of the genomic target sequence may have been too large for amplification. To select for specific fragments, we discarded amplicons where agarose gel electrophoresis showed more than a single fragment. The average fragment size of amplicons was approximately 600 bp. The ESTs and GSS fulfilling the above criteria were sequenced (Additional File 2). The 82% of the 100 EST-derived amplicons and 77% of 100 GSS-derived amplicons were successfully sequenced. Most of the amplicons from *A. duranensis* and *A. ipaensis* were homozygous and the homozygosity of EST-derived amplicons (87.8%) was higher than that of GSS-derived amplicons (75.3%). On the contrary, most of the amplicons from *A. hypogaea* were heterozygous and the heterozygosity of GSS-derived amplicons (76.6%) was higher than that of EST-derived amplicons (62.2%).

**Figure 1:**
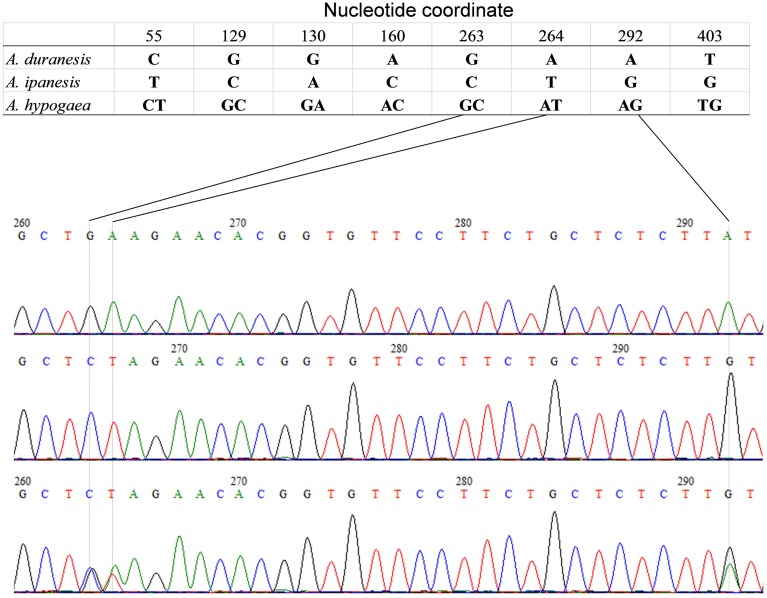
**Amplicon Sequence of EST-48 in *A. hypogaea* and putative diploid progenitor**. The figure shows that the *A. duranensis* and *A. ipaensis* genomes each contained single copy of sequence “EST-48” while both the copies were retained in *A. hypogaea* genome.

**Figure 2:**
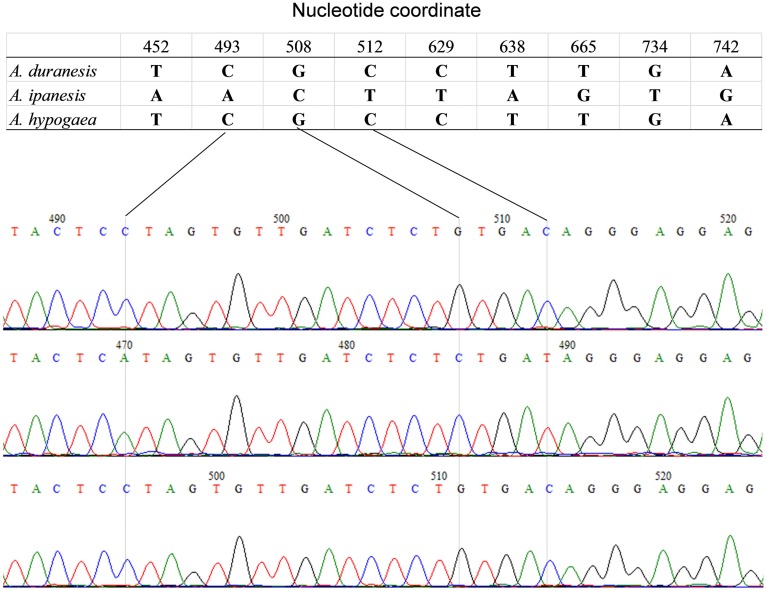
**Amplicon Sequence of EST-86 in *A. hypogaea* and putative diploid progenitor**. The figure shows that the *A. duranensis* and *A. ipaensis* genomes each contained single copy of sequence “EST-86” while single copy from *A. duranensis* genome retained in *A. hypogaea* genome.

**Figure 3:**
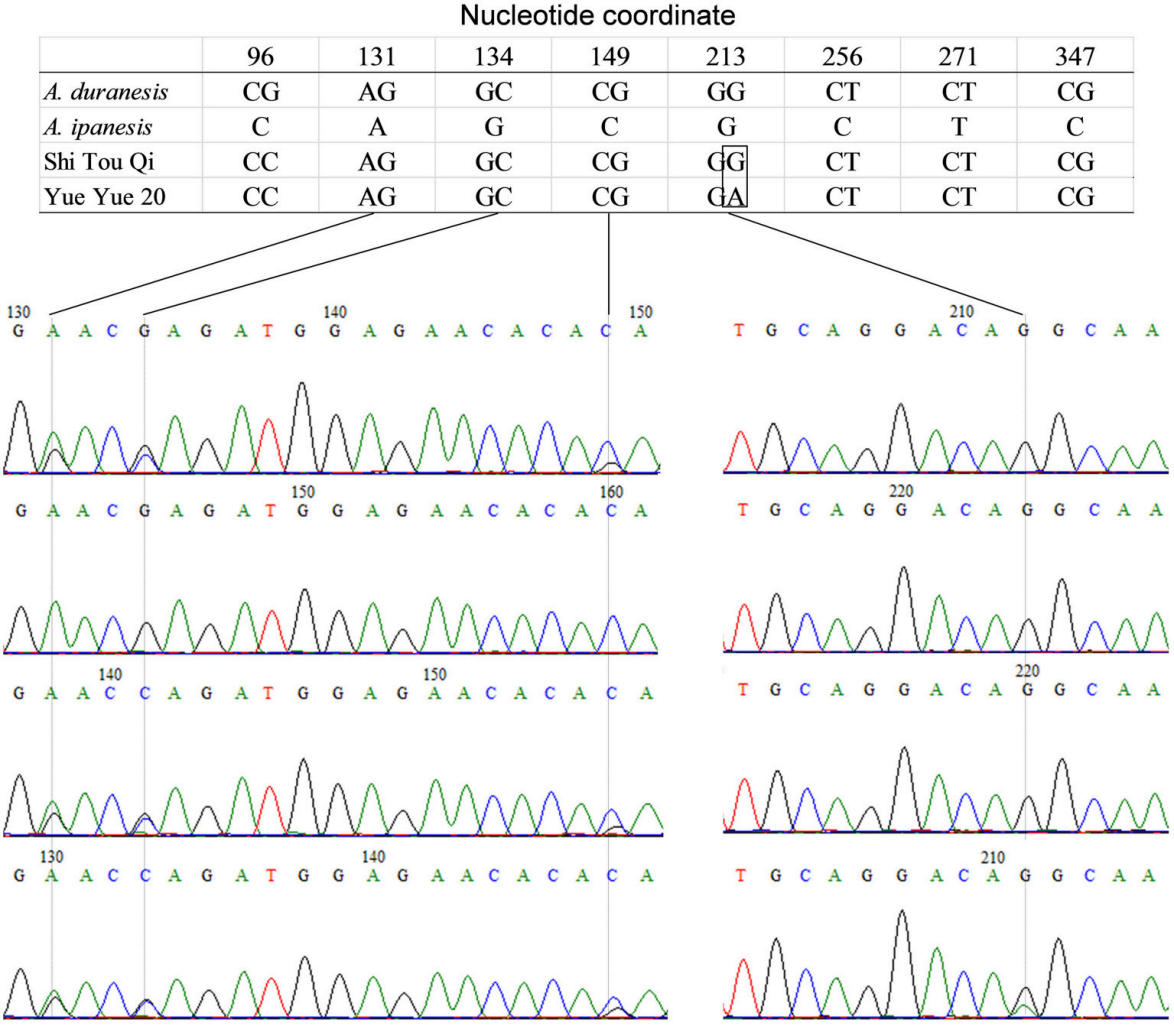
**Amplicon sequence of EST-87 in *A. hypogaea* and putative diploid progenitor**. The figure shows that the *A. duranensis* genome contained two copies of sequence “EST-87” while *A. ipaensis* genome had only single copy. The single copy from *A. duranensis* genome was retained in *A. hypogaea* genome.

The detection of heterozygous amplicons in diploid progenitors indicated that the PCR fragments contain paralogous sequences. The co-existence of A and B genomes in *A. hypogaea* made the composition of heterozygous amplicon more complicated. It may contain homoeologous and (or) paralogous sequence. Sequence alignment between *A. hypogaea* and diploid progenitors showed that most of the PCR fragments in *A. hypogaea* were derived from both A and B genome (Figure 1), but some were derived from only one of the subgenomes. The sequence EST-86 existed in A genome but was absent in B genome (Figure 2). The detection of paralogous sequences and DNA sequence elimination in the early stage after the formation of allopolyploids (Adams and Wendel, 2005) increased the difficulty in identifying SNPs, HSVs, and PSVs. However, by sequence alignment of *A. hypogaea* with diploid progenitors, different sequence variation categories can be distinguished. It was also found that both the copies of EST-87 were derived from A-genome (Figure 3), hence the sequence variation between the copies were PSVs rather than HSVs.

### Frequency of SNPs, HSVs, and PSVs

Of the 82 sequenced EST-derived amplicons, only 12 were polymorphic in the 96 varieties (i.e., they contained at least 1 SNP), yielding a total of 18 SNPs averaging one SNP in every 2557 bp in the 12 peanut varieties (Additional File 3). SNPs in the nucleotide variant classes C/T (G/A) and A/C (T/G) were most common (both were 38.9%), while A/T (T/A) and C/G (G/C) variants were less prevalent (16.7 and 5.5%, respectively). Of the 77 sequenced GSS-derived amplicons, 20 were polymorphic, yielding a total of 44 genomic-SNPs in the investigated panel (Additional File 4). The frequency of genomic-SNPs was one SNP/1011 bp, which was much higher than that of the EST-SNPs. Nucleotide variant classes of genomic-SNPs was similar to that of EST-SNPs i.e., C/T (G/A) and A/C (T/G) were most common with a frequency of 68.2 and 22.7%, respectively, while A/T (T/A) and C/G (G/C) were the least prevalent with a frequency of 6.8 and 2.3%, respectively.

A total of 306 EST-HSVs were identified in *A. hypogaea*, which were distributed in 43 heterozygous EST-derived amplicons with one SNP in every 76.9 bp. EST-HSVs in the nucleotide variant classes C/T (G/A) were most common (71.6%), while C/G (G/C) (13.7%), A/C (T/G) (7.8%), and A/T (T/A) (6.9%) variants were less prevalent. Meanwhile, 869 genomic-HSVs were identified from 48 heterozygous GSS-derived amplicons. The frequency of genomic-HSV was higher than that of EST-HSVs, averaging one SNP in every 32.2 bp. Genomic-HSVs in nucleotide variant classes C/T (G/A) were most common (61.4%), while A/C (T/G), A/T (T/A), and C/G (G/C) were less prevalent with18.1, 15.8, and 4.6%, respectively.

Total eight EST-derived amplicons containing 54 PSVs were identified in *A. hypogaea*, averaging one SNP in every 86.8 bp, while 11 GSS-derived amplicons containing 277 PSVs were identified in *A. hypogaea*, averaging one SNP in every 27 bp. Nucleotide variant classes of EST-PSVs and genomic-PSVs were similar. The C/T (G/A) was most common, while A/C (T/G), A/T (T/A) and C/G (G/C) were less prevalent.

### Allele frequency

**Figure 4:**
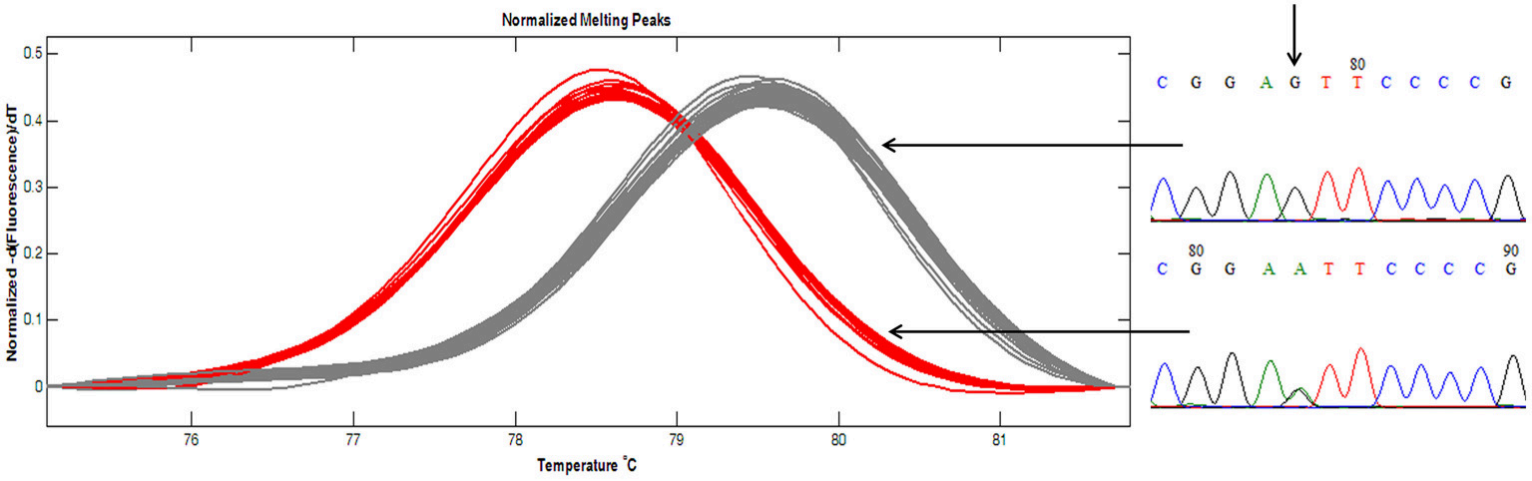
**Amplicon melting analysis of ampilcon EST-66**. A total of 96 peanut varieties were analyzed by HRM in ampilcon EST-66. Of the 96 varieties, 28 samples showed red line, and remaining 68 samples showed in gray lines. Representative sequence traces were shown on the right; homozygote at the top and heterozygote at the bottom. Vertical arrows showed the SNP position.

Thirty two SNP-containing fragments were available for analyzing the panel of 96 peanut varieties of south China. For 30 fragments, one SNP was enough for genotyping since 20 fragments contained only one SNP and the other 10 fragments contained more than one SNP showed fully linkage disequilibrium (*r*^2^ = 1). For GSS-56 and GSS-67, both *r*^2^ < 1, two SNPs were determined. Therefore, 34 SNPs were available for PIC estimation in the 96 peanut varieties. A total of 34 nest primers were used for PCR amplification and HRM analysis. The results indicated that except GSS-4, HRM melting curve of the other 33 amplicons were well distinguished in the investigated panel, and the genotypes identified by HRM analysis was consistent with that by amplicon sequencing (Figure 4). SNPs are typically bi-allelic, therefore, the PIC values for single SNP cannot be greater than 0.5. PIC values for EST-SNP markers in peanut ranged between 0.021 and 0.413 with a mean of 0.172 (Table 1). PIC values for GSS-SNPs were higher than EST-SNPs in peanut, which ranged between 0.080 and 0.478 with a mean of 0.249.

**Table 1:** **Allele frequencies and PIC values of SNP markers in peanut**.

**Marker**	**Primer sequence**		**Variant classes**	**Allele frequency**		**Polymorphic information content (PIC)**
	**Forward**	**Reverse**		**Allele 1 (%)**	**Allele 2 (%)**	
EST-11SNP	TTCACGCCAGATGAGGAT	CAGGAGTCGGGCTATTGT	C/G	4	92	0.080
EST-12SNP	CATCTCAGCACTCAACTT	GAGTAAGGGTTTGAGGAA	C/T	1	95	0.021
EST-21SNP	TTGAGTGAACCAGCTTGAGG	GCCGACGAAGAAGAGGAATA	G/T	15	81	0.264
EST-26SNP	TACCCTTGACTTGGTTTATGGC	GCTCTTCAGCGAACTGTCCC	A/C	8	88	0.153
EST-33SNP	CAAACAAAGTCATCGCAGTC	CTCTTCTATCCCATCTCACAG	G/T	92	4	0.080
EST-45SNP	AACTGGCGTCTTCTTCACCG	GTCCCAAGCCTGCATCCAAA	G/T	88	8	0.153
EST-48SNP	ACCGCTTACTATCACTACCA	TCTAATATGACTGTTCCCAGA	G/T	83	13	0.234
EST-66SNP	CATCGTCATCCGTGAGGTGT	TTGGACTTGAGGAGGAGGTAG	A/G	28	68	0.413
EST-70SNP	AGGCGTTGAAATGCAGGTCC	GGCATCCCAGTTCCAGAAGG	C/T	79	17	0.291
EST-80SNP	ACAGAGTCATTGGTGATGGGAGTT	CACTTGCAGGTGCATGGGTT	A/G	5	91	0.099
EST-87SNP	ATGAGCTGGACCAGATGGAG	CCTGAAGTTACACTGTTGAGGC	A/G	13	83	0.234
EST-92SNP	GAGTAATCGTTGAGAAACTCGC	TCAGAAGACTTCGCCACCTT	A/T	2	94	0.041
GSS-2SNP	ACTTAGCATAAGAGGGTATTTG	AGCGGAACAGGATAAGCAAT	A/G	14	82	0.249
GSS-10SNP	GCCAGAACTATGCTTCCCTT	AAATCCCTCTGAGGACAATC	A/C	21	75	0.234
GSS-26SNP	GACTCAGCGCAGTGCCAACT	GTGCCATGCTTACTCAACAAAA	C/A	85	11	0.203
GSS-35SNP	ATTGAACAAAGGATTGAGAT	CAAATGAAGGTGTAGATGGC	A/T	24	72	0.375
GSS-44SNP	CAACATCCAGACACCCCAAAT	CTCCAGGTCAGGCTGAACAT	A/C	16	80	0.278
GSS-45SNP	TCTAGCCAGATCGGCCATAC	GAATACGTGACTGACCCAAG	A/G	87	9	0.170
GSS-46SNP	TCTACCTTTGCCTTATCCAC	ACTTGTTCTGAAAAGATGCC	C/T	4	92	0.080
GSS-49SNP	TTCAATGCTCAATCATTCCCACTA	GAGGACTTGTCCGCCACCTT	A/C	18	78	0.305
GSS-52SNP	AAGCCTTAAACAGGGGTAGC	TTGGTAGGAGGTGAGCGATG	C/T	89	7	0.135
GSS-54SNP	AGTGCAAAGGTTGCGTCTTG	TTGCTCCATTTTCATTAGGTTTT	C/T	82	14	0.249
GSS-56-1SNP	ACATTGGCAGGTGGTGGTAA	ATGGGAGAAATGTCACTTTATGGT	A/G	6	90	0.117
GSS-56-2SNP	TTCTGTATGCAAATACTCCG	ATGAATACTACTGCTTCCTG	C/T	15	81	0.264
GSS-67-1SNP	ATAATCCACTGCCACCAGAA	TATGCTATGCCTCAACAAGG	C/T	87	9	0.170
GSS-67-2SNP	ATGGTTCATCCCAAAGATAG	TGAGATTCATACCCAAAGAG	C/T	83	13	0.234
GSS-69SNP	TGGCTAGAGGATGGTTGGAG	TGATAGCCCGGTCTATGGTA	A/C	26	70	0.395
GSS-72SNP	CATTCTACTGGTGGGTCTGT	CATGAACTGGATTATTTGCA	A/T	71	15	0.429
GSS-74SNP	ATTTGCCACTAACTCCCTTC	TTAGCATACAAGATAGTTGAAAGT	C/T	89	7	0.135
GSS-76SNP	ACAAGCTATCCCAACTCCAC	TAAGCAGTCCACCAATCAAA	A/G	33	61	0.478
GSS-80SNP	CCTAGAATTATTCATAACCTCCAT	GCTTACACGTCACATGCTTT	A/G	17	79	0.291
GSS-81SNP	CCATTACTTGTTTTGCTCAC	TGTGGTTATAGAACAGAGGG	C/T	83	13	0.234
GSS-99SNP	TAGAAAGCCCTGGATGTTAG	AAAGAGTGCAGATGCTGTCA	C/T	90	6	0.117

### Genetic mapping of SNPs

**Figure 5:**
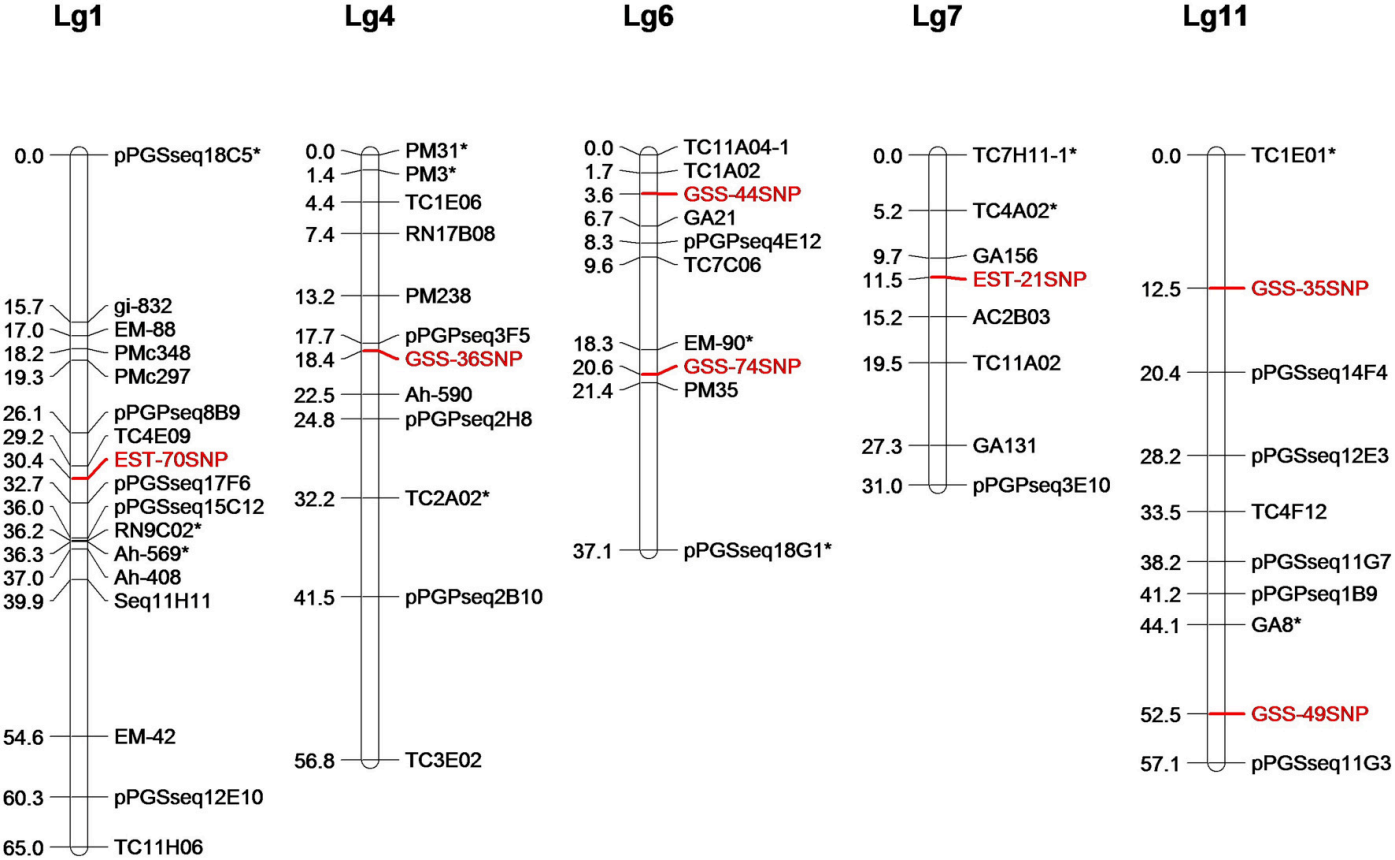
**Location of seven SNPs markers on LGs of RIL population Y13Zh**. Seven SNPs markers were mapped into five linkage groups of RIL population Y13Zh, which were showed in red. ^*^Indicates distorted marker loci.

Of the 33 SNPs used for HRM analysis, nine showed polymorphism between Zhenzhuhei and Yueyou13. All the nine SNP loci segregated in the mapping population Y13Zh and two loci showed segregation distortion. Both the distorted loci were in favor of Y13 alleles. Through linkage analysis with the SSR marker for population Y13Zh, seven SNP loci were successfully mapped to five linkage groups of population Y13Zh (Figure 5).

## Discussion

The SNP identification in an allopolyploid species such as *A. hypogaea* is very complex and difficult as several issues have to be taken into account in identifying good quality SNPs in such crops. One of the challenging tasks in this approach was to identify reliable SNPs that can actually be used for genotyping mapping populations and individual varieties. Firstly, the SNPs need to be identified that are indeed polymorphic between individual varieties, meaning that they must be polymorphic at a given position in only one of the two ancestral genomes. Such SNPs must be discriminated from SNPs that are present between the A and B genomes of the same genotype (*A. hypogaea*) and (or) between closely related copies of genes that are members of gene families. Methods that rely on the comparison of ESTs and GSS from different varieties face a bioinformatic challenge, because SNPs between paralogous sequences and the transcripts from the two genomes have to be clearly identified. In order to achieve good results by keeping all the above considerations, a very high redundancy of sequences are required from each of the identified varieties. The available ESTs and GSS in the NCBI database are at present not providing the necessary sequence depth. The Zhou et al. (2014) has recently approached this problem by massively parallel sequencing of the *A. hypogaea* genome using the Illumina NGS platform. This approach has led to the identification of more than 53, 257 putative SNPs. However, only a limited number of the identified SNPs were experimentally validated and used for constructing the genetic linkage map. Hence, it is not fully clear what proportion of the identified SNPs might be false positives. Other complexity reduction technologies will most likely face similar problems and probably result in a considerable proportion of false SNPs, raising the costs for SNP validation.

The SNPs prevalence in *A. hypoaea* is low in comparison to other inbreeding plant species such as *A. thaliana*, soybean and wheat (Jander et al., 2002; Zhu et al., 2003; Russell et al., 2004). However, the frequencies of HSVs and PSVs in *A. hypogaea* were significantly higher than that of SNPs, even were dozen times of SNPs. Most importantly, once homoeologous and paralogous sequences were misidentified as homologous sequences, it will significantly decrease the validation rate of SNPs. Thus, when NGS is used to identify SNP in *A. hypogaea*, sequence alignment under conditions of high stringency using appropriate alignment parameters such as high threshold for minimum match percentage and minimum overlap, will reduce coalescence of homoeologous, paralogous and homologous sequences, and finally increase the validation rate of SNPs.

In this study, we have used amplicon sequencing in a panel of peanut varieties and used samples of the ancestral genomes for the identification of SNPs based on peanut ESTs and GSS. The amplicon sequencing approach that has been presented here is a complex approach that includes the sequencing of individual amplicons generated from individual genes, requiring considerable work. The advantage of this approach, especially in an allopolyploid species, lies in the fact that only truly unique and well characterized sequences are being analyzed on the two genomes. Amplicons that amplify more than one locus in one or both genomes will most likely fail during sequencing, as the group of fragments will most likely contain InDels so that no readable sequence is produced. This is an efficient filtration step. It could be demonstrated that fragments which are derived from only one locus in each of the genomes can, in most cases, be sequenced in high quality from at least one side. The sequencing of such products from a number of lines, in combination with the ancestral genomes, permits reliable identification and classification of SNPs. However, since the frequency of SNPs in *A. hypogaea* is low, identification SNPs only by amplicon sequencing will cause low efficency. To improve the efficency and validation rate of SNP in *A. hypogaea*, combining NGS and amplicon sequencing will be an efficient approach. In other words, the candidate SNPs will be identified using the large scale NGS data and then validation of these SNPs will be done by amplicon.

Evolutionary studies have suggested elimination of DNA sequence in the early stage after the formation of allopolyploids and was observed more for the multiple-copy sequences (Rieseberg, 2001; Renny-Byfield et al., 2012). The elimination of sequences increased the divergence between two homoeologous chromosomes providing a physical basis for rapid restoration of diploid-like chromosome paring pattern during meiosis. In the present study, sequence alignment of amplicons between diploid progenitor and cultivated tetraploid (*A. hypogaea*) verified that cultivated tetraploid peanut has also undergone DNA sequence elimination in its early stage of formation. The sequencing traces indicated that multiple-copy sequences were more easily eliminated than the single-copy sequence in *A. hyopgaea* genome, similar to other species (Ma et al., 2004; Tang et al., 2008).

As a positive development, recently few studies in plants demonstrated utility of HRM in detection of genetic variation and genotyping (Chateigner-Boutin and Small, 2007; Lehmensiek et al., 2008; Wu et al., 2008). It has been observed that the HRM analysis is able to detect all single base changes with greater sensitivity for G/A and C/T changes, and lower sensitivity for A/T and G/C changes (Liew et al., 2004). In our study, the nucleotide variant classes C/T (G/A) in SNPs of peanut were most prevalent. Considering all the above facts, HRM is very efficient approach and is suitable for SNP genotyping in peanut.

In the present study, the frequency and PIC value of genomic-SNPs was significantly higher than the EST-SNPs in *A. hypogaea*. The results indicate that the genomic-SNPs are distributed widely and evenly in the peanut genome of *A. hypogaea* and, therefore, the genomic-SNPs will be more efficient than EST-SNPs in genetic mapping, association analysis and in other breeding applications. In this study, nine SNP markers showed polymorphism in the RIL population Y13Zh, and two SNPs cannot be mapped into the linkage groups of peanut. Since the two SNP markers did not show segregation distortion in the mapping population, it can be inferred that SNP genotyping was correctly completed by HRM, and their long linkage distance with the other markers may have caused the failure of mapping into the linkage groups.

### Conflict of interest statement

The authors declare that the research was conducted in the absence of any commercial or financial relationships that could be construed as a potential conflict of interest.

## References

[B1] Adams K. L. Wendel J. F. (2005). Polyploidy and genome evolution in plants. Curr. Opin. Plant Biol. 8, 135–141. 10.1016/j.pbi.2005.01.001 15752992

[B2] Anderson J. A. Churchill G. A. Autrique J. E. Tanksley S. D. Sorrells M. E. (1993). Optimizing parental selection for genetic linkage maps. Genome 336, 181–186. 10.1139/g93-024 18469981

[B3] Bertioli D. J. Ozias-Akins P. Chu Y. Dantas K. M. Santos S. P. Gouvea E.. (2014). The use of SNP markers for linkage mapping in diploid and tetraploid peanuts. G3 (Bethesda) 4, 89–96. 10.1534/g3.113.007617 24212082 PMC3887543

[B4] Botstein D. White R. L. Skolnick M. Davis R. W. (1980). Construction of a genetic linkage map in man using restriction fragment length polymorphisms. Am. J. Hum. Genet. 32, 314–331. 6247908 PMC1686077

[B5] Chang C. C. Chang Y. S. Chan W. L. Yeh K. T. Wei R. J. Chang J. G. (2014). Detection of *SF3B3* gene mutations in oral cancer by high resolution melting analysis. Clin. Lab. 60, 2023–2029. 10.7754/Clin.Lab.2014.140409 25651737

[B6] Chateigner-Boutin A. L. Small I. (2007). A rapid high-throughput method for the detection and quantification of RNA editing based on high-resolution melting of amplicons. Nucleic Acids Res. 35, E114. 10.1093/nar/gkm640 17726051 PMC2034463

[B7] Cho M. H. Ciulla D. Klanderman B. J. Raby B. A. Silverman E. K. (2008). High-resolution melting curve analysis of genomic and whole-genome amplified DNA. Clin. Chem. 54, 2055–2058. 10.1373/clinchem.2008.109744 19042988 PMC2755063

[B8] Croxford A. E. Rogers T. Caligari P. D. S. Wilkinson M. J. (2008). High-resolution melt analysis to identify and map sequence-tagged site anchor points onto linkage maps: a white lupin (*Lupinus albus*) map as an exemplar. New Phytol. 180, 594–607. 10.1111/j.1469-8137.2008.02588.x 18684160

[B9] Erali M. Voelkerding K. V. Wittwer C. T. (2008). High resolution melting applications for clinical laboratory medicine. Exp. Mol. Pathol. 85, 50–58. 10.1016/j.yexmp.2008.03.012 18502416 PMC2606052

[B10] Fredman D. White S. J. Potter S. Eichler E. E. Den Dunnen J. T. Brookes A. J. (2004). Complex SNP-related sequence variation in segmental genome duplications. Nat. Genet. 36, 861–866. 10.1038/ng1401 15247918

[B11] Ganal M. W. Altmann T. Röder M. S. (2009). SNP identification in crop plants. Curr. Opin. Plant Biol. 12, 211–217. 10.1016/j.pbi.2008.12.009 19186095

[B12] Guo B. Chen X. Dang P. Scully B. T. Liang X. Holbrook C. C.. (2008). Peanut gene expression profiling in developing seeds at different reproduction stages during *Aspergillus parasiticus* infection. BMC Dev. Biol. 8:12. 10.1186/1471-213X-8-12 18248674 PMC2257936

[B13] Hammons R. O. (1994). The origin and early history of the groundut in The Groundnut Crop: A Scientific Basis for Improvement, ed Smartt J. (London, Chapman and Hall press), 24–42.

[B14] Hand M. L. Ponting R. C. Drayton M. C. Lawless K. A. Cogan N. O. Charles Brummer E.. (2008). Identification of homologous, homoeologous and paralogous sequence variants in an outbreeding allopolyploid species based on comparison with progenitor taxa. Mol. Genet. Genomics 280, 293–304. 10.1007/s00438-008-0365-y 18642031

[B15] Hong Y. Chen X. Liang X. Liu H. Zhou G. Li S.. (2010). A SSR-based composite genetic linkage map for the cultivated peanut (*Arachis hypogaea* L.) genome. BMC Plant Biol. 10:17. 10.1186/1471-2229-10-17 20105299 PMC2835713

[B16] Jander G. Norris S. R. Rounsley S. D. Bush D. F. Levin I. M. Last R. L. (2002). *Arabidopsis* map-based cloning in the post-genome era. Plant Physiol. 129, 440–450. 10.1104/pp.003533 12068090 PMC1540230

[B17] Kaur S. Francki M. G. Forster J. W. (2012). Identification, characterization and interpretation of single-nucleotide sequence variation in allopolyploid crop species. Plant Biotechnol. J. 10, 125–138. 10.1111/j.1467-7652.2011.00644.x 21831136

[B18] Kristensen L. S. Mikeska T. Krypuy M. Dobrovic A. (2008). Sensitive Melting Analysis after Real Time-Methylation Specific PCR (SMARTMSP): high-throughput and probe-free quantitative DNA methylation detection. Nucleic Acids Res. 36:E42. 10.1093/nar/gkn113 18344521 PMC2367707

[B19] Lehmensiek A. Sutherland M. W. McNamara R. B. (2008). The use of high resolution melting (HRM) to map single nucleotide polymorphism markers linked to a covered smut resistance gene in barley. Theor. Appl. Genet. 117, 721–728. 10.1007/s00122-008-0813-4 18553067

[B20] Liew M. Pryor R. Palais R. Meadows C. Erali M. Lyon E.. (2004). Genotyping of single-nucleotide polymorphisms by high-resolution melting of small amplicons. Clin. Chem. 50, 1156–1164. 10.1373/clinchem.2004.032136 15229148

[B21] Ma X. F. Fang P. Gustafson J. P. (2004). Polyploidization-induced genome variation in triticale. Genome 47, 839–848. 10.1139/g04-051 15499398

[B22] Mastoraki S. Chimonidou M. Dimitrakopoulos L. Kounelis S. Malamos N. Georgoulias V.. (2015). A rapid and accurate closed-tube Methylation-Sensitive High Resolution Melting Analysis assay for the semi-quantitative determination of *SOX17* promoter methylation in clinical samples. Clin Chim. Acta 444, 303–309. 10.1016/j.cca.2015.02.035 25727515

[B23] Minton J. A. Flanagan S. E. Ellard S. (2011). Mutation surveyor: software for DNA sequence analysis. Methods Mol. Biol. 688, 143–153. 10.1007/978-1-60761-947-5_10 20938837

[B24] Monis P. T. Giglio S. Saint C. P. (2005). Comparison of SYT09 and SYBR Green I for real-time polymerase chain reaction and investigation of the effect of dye concentration on amplification and DNA melting curve analysis. Anal. Biochem. 340, 24–34. 10.1016/j.ab.2005.01.046 15802126

[B25] Moretzsohn M. C. Leoi L. Proite K. Guimarães P. M. Leal-Bertioli S. C. Gimenes M. A.. (2005). A microsatellite-based, gene-rich linkage map for the AA genome of *Arachis* (Fabaceae). Theor. Appl. Genet. 1, 1060–1071. 10.1007/s00122-005-0028-x 16088397

[B26] Nagy E. D. Guo Y. Tang S. Bowers J. E. Okashah R. A. Taylor C. A.. (2012). A high-density genetic map of *Arachis duranensis*, a diploid ancestor of cultivated peanut. BMC Genomics 13:469. 10.1186/1471-2164-13-469 22967170 PMC3542255

[B27] Pandey M. K. Monyo E. Ozias-Akins P. Liang X. Guimarães P. Nigam S. N.. (2012). Advances in *Arachis* genomics for peanut improvement. Biotechnol. Adv. 30, 639–651. 10.1016/j.biotechadv.2011.11.001 22094114

[B28] Parkin I. A. P. Sharpe A. G. Keith D. J. Lydiate D. J. (1995). Identification of the A and C genomes of amphidiploid *Brassica napus* (oilseed rape). Genome 38, 1122–1131. 10.1139/g95-149 18470236

[B29] Renny-Byfield S. Kovařík A. Chester M. Nichols R. A. Macas J. Novák P.. (2012). Independent, rapid and targeted loss of highly repetitive DNA in natural and synthetic allopolyploids of *Nicotiana tabacum*. PLoS ONE 7:e36963. 10.1371/journal.pone.0036963 22606317 PMC3351487

[B30] Rieseberg L. H. (2001). Polyploid evolution: keeping the peace at genomic reunions. Curr. Biol. 11, 925–928. 10.1016/S0960-9822(01)00556-5 11719240

[B31] Russell J. Booth A. Fuller J. Harrower B. Hedley P. Machray G.. (2004). A comparison of sequence-based polymorphism and haplotype content in transcribed and anonymous regions of the barley genome. Genome 47, 389–398. 10.1139/g03-125 15060592

[B32] Seijo J. G. Lavia G. I. Fernández A. Krapovickas A. Ducasse D. Moscone E. A. (2004). Physical mapping of the 5S and 18S-25S rRNA genes by FISH as evidence that *Arachis duranensis* and *A. ipaensis* are the wild diploid progenitors of *A. hypogaea (Leguminosae)*. Am. J. Bot. 91, 1294–1303. 10.3732/ajb.91.9.1294 21652361

[B33] Somers D. J. Kirkpatrick R. Moniwa M. Walsh A. (2003). Mining single-nucleotide polymorphisms from hexaploid wheat ESTs. Genome 49, 431–437. 10.1139/g03-027 12834059

[B34] Tang Z. X. Fu S. L. Ren Z. L. Zhou J. P. Yan B. J. Zhang H. Q. (2008). Variations of tandem repeat, regulatory element, and promoter regions revealed by wheat-rye amphiploids. Genome 51, 399–408. 10.1139/G08-027 18521118

[B35] Varshney R. K. Mohan S. M. Gaur P. M. Gangarao N. V. Pandey M. K. Bohra A.. (2013). Achievements and prospects of genomics-assisted breeding in three legume crops of the semi-arid tropics. Biotechnol. Adv. 31, 1120–1134. 10.1016/j.biotechadv.2013.01.001 23313999

[B36] Vossen R. H. Aten E. Roos A. den Dunnen J. T. (2009). High-resolution melting analysis (HRMA): more than just sequence variant screening. Hum. Mutat. 30, 860–866. 10.1002/humu.21019 19418555

[B37] Wittwer C. T. Reed G. H. Gundry C. N. Vandersteen J. G. Pryor R. J. (2003). High-resolution genotyping by amplicon melting analysis using LCGreen. Clin. Chem. 49, 853–860. 10.1373/49.6.853 12765979

[B38] Wu S. B. Wirthensohn M. Hunt P. Gibson J. Sedgley M. (2008). High resolution melting analysis of almond SNPs derived from ESTs. Theor. Appl. Genet. 118, 1–14. 10.1007/s00122-008-0870-8 18781291

[B39] Zhou X. Xia Y. Ren X. Chen Y. Huang L. Huang S.. (2014). Construction of a SNP-based genetic linkage map in cultivated peanut based on large scale marker development using next-generation double-digest restriction-site-associated DNA sequencing (ddRADseq). BMC Genomics 15:351. 10.1186/1471-2164-15-351 24885639 PMC4035077

[B40] Zhu Y. L. Song Q. J. Hyten D. L. Van Tassell C. P. Matukumalli L. K. Grimm D. R.. (2003). Single-nucleotide polymorphisms in soybean. Genetics 163, 1123–1134. Available online at: http://www.genetics.org/content/163/3/1123.full#ref-list-1 12663549 10.1093/genetics/163.3.1123 PMC1462490

