# Supplementary material for: Identification and Evaluation of Single-Nucleotide Polymorphisms in Allotetraploid Peanut (Arachis hypogaea L.) Based on Amplicon Sequencing Combined with High Resolution Melting (HRM) Analysis
Source: Front Plant Sci. 2015 Dec 2;6:1068. doi: 10.3389/fpls.2015.01068 (PMC4667090; doi:10.3389/fpls.2015.01068)
Supplement: Supplementary file 3 [file DataSheet3.DOC]

**Additional File 3: List of EST-SNPs identified in *A. hypogaea***

Description: – Eighteen EST-SNPs were identified and twelve were used for genotyping. SNP used for genotyping were shaded in yellow.

EST-11

>ALB-2-13E_12-5'-TripE-SP

AAAAAACCCTTTCAAAAATCTCATCGTAACGCCTTACCTACTTCGCTTCTGTTTCTCTCCATATATAAATCTTCACTTTCACATGATCCTCTAAATCTGAAAAACAATACAAAGGAAGCTAAGCAGTATGGCCATGTCTAGAAGAGAAATGGATCGGATCAAAGGCCCGTGGAGCCCGGAAGAAGATGAGGCTCTGCAGAGGCTGGTGGAGAGGCACGGGCCCAGGAACTGGACGTTGATAAGCAAGTACATCCCGGGCCGCTCCGGGAAGTCCTGCAGGCTAAGATGGTGTAACCAAC**T/G**GTCACCTCAGGTTGAGCACCGGGGCTTCACGCCAGATGAGGATGAGACGATAATCCGGGC**C/G**CATGCCCGGTTCGGTAACAAGTGGGCTACAATAGCCCGACTCCTGTCTGGTCGAACTGACAACGCGGTAAAGAATCACTGGAACTCAACACTCAAGCGCAAGTGCTCATCCATGATGATCGACGAGCCTGCAACAGGTCCGGCAGACTTTGACGGCGGTTACTACCTACGGCCCGCGAAGAGGTCTGCGAGCGCAGGTGCTACCATGCCGCTATCCACCGGGCTTCACATGAGCCCGACGTCGCCGGGAAGCCCGTCGGGATCCGACGCGAGTGATTGCAGCGTTCGCGCTGTCTCTCCGTCGCACGTTCTGAGGCCCGTGCAGAGGACCGTTGTTCCTCCGATCGAAACTGCGTCCTCTTCAGAGGACCCTCCGACGTCGTTGTCGCTGTCTCTGCCTGGCGTAGACGTTGCTGTGGAAGTCTCCAATCGTGTCACGGAATCTATGTCACGTGCCTTTTCTCCGGCGA

EST-12

>SunSeed_CL862Contig1

CCATCCTTGAAATGGTGAAAACGATTTGTGTCTCTTACAAAAATTTCTCTCCCTTCCAAACTGCTAATAAATTTTATAACGGCATGGCAGTCACTGCAGATTCTCAGGTTCTTAATCACTTGAAG**A/T**GGCTGCCCCGGACTCGTGTTGAGTAGTCCTAATACTACTGCCAACTTCTCACTGTGCCCACACAATATCTGCTCCTTCTCTTGTTCCTCCACATCGTGCAGCACAAGTCCAGTCTTAGGAAGGTGACCACATTTCTTCATCTCAGCACTCAACTTTTCCAATTTCTCCAATATCTCTTGCATTTGTGGATG**C/T**GACTTGTCACCAGCAACAAGCATGTGTACTTTGTTTCCAATCTCAATCCAACTGTAGCCAGGATTTTTCCTCAAACCCTTACTCTTCATCACATCACGAATTCTGTCTACTTCATCCCACATGCCCTTTGAAGCATAGATGTTGGACAGAAGAATGTAGTTCCCCGGGTTATCGGGTTCTAACAGGAAAAGCTTTTCTGCAGCAATCTCGCCTAAACTCAAATTATGGTGAACTCTACAGGAACTTAGTAAGGCTCCCCAGATGCAAGCATCAGGTTCTAATGGCATTTCCTTGATGAGAGAATAAGCTTCTTCAAGTTTCCCTACACGACTAAGAAGTGTCACCATGCAAGCATAGTGTTCTGTTTTAGTTTCAATGGCATGTTCTTTTGACAGGCTATTGAAATATTGCCACCCTTCTTCAGTTAAGCCATTTTGGGCGCATGCGTATAACACACATGTGTAGGAAATCTCATCAGGCTCCATGCCACTCTCTTGCATCATTCGAAACATCTCTATTGTTTCCTTAACCTTCCCATGCATTGCAT

EST-21

>SunSeed_CL10Contig3

TTTTTTTTTTTTTTTTTTTTTTTTTTTTTTCTTTTTTTTTTTTTTTTTTTTTTTTTTTTTTTTTTTTTTTTTTTTTTTGCGTTAAATCATCCGTTCTTTTATTCATGTTTTTCCCGCAAAAGGTTTTCTCCAGATCGATGCACATACATTACATTCCGATCTTATAGCTTAAACAAAAAGGCATGAATGCCCTTTTGTATTCTTATTGACACTACAAAATTAAAACTACATTATTCTCGGTCGTTATTAATATGTATTATTCAGTAGTTGTGGCAAAAAGTATGTATGTAGTGGATAACACACATACATGTCACGCTTGTTTTCAAGCCACAGCCCTGAGAGACTGTTGAGACGGTGGAACGAAGAACTTGAAGGGGTTGTTGTTCTTAAGCTGCCTTGCCTGCTCCCTTGGGAGGCCATATGAATTTGCAACCACCTCCTCCGGCAAGTTATCTATGATGGAGTTTTCACCGGCGAGGTTGGCTATGCTGGGCCTTGAGTCTGTCTTGAATGCCACGTATTCAAAGTTGTCGCTCTGGGACTTTCCAGCGACGGCGAAGTTCTGTGGCACCACAAGCACGTGACCCTCTTGAAGCTCCTCGTCGTACACTCTGTTGCCGTTGCTGTCCACGACTTGCACGTGAGCCCGTCCCCTCAATGCATATATGATGCTGTGTGCGTTGGTGTTGTAGTGAGGGACAAACAATGCATTCCTGTAGAGATTTCCATATTCAGCACTAAGTCCAAGCCACCTAAGGATTAGAAGGTTGAGATCGTTGGCAGTTTTGAGTGAACCAGCTTGAGGATTGTAGATGTCAGGGGATCTGTTTCTACCAATGTTCTTTTTAACACTTGC**T/G**GTGCAGATCGTCTCTTCAATACCATTCCCCCTGCCTCTGCTTCCCCTGCCACGCCTTCTATCCTCTTCATCGTATTCATATTCATCTTCATCGTATTCCTCTTCTTCGTCGGCACCTCTCTTTCTATCTGGGCTCAAGATTCTGAGGCCTCCCTTCACTGTCACAATGGCTCCCTCTTCCTCACTCTCGTTCTCGCCTCTTAGATTTTGCACTATCTGTCTGTCGTCAACCTGGAAGGCTTGTGCCAGGAACTCCGGCGTGAAGCCGCTGAAGATGTTTCCACCTTCGTTTTCTTCTTCTTGTCCTGCTCGTTCTCTGCGGCTGTGCTGTCCTCGAGGGCTAAATTCACGCTCTTCTTGTCTAGGCTGACTTTGCGGGCTGTATGGGCTATATGGTAAGCTTCTTCGTCTGCTTTGTTGCTGGTATCTTAAGAACTCTTGCTCGTGGTTTCCAGCCAAATTGAATCTCCTGGGGAACTGATCAAGCTGGTTGTCGTTGTTGTTGGTGTCAGTAAGAGAAACAGCAACAACATCAGTGTCATGGTCGTTGTACATCCAGAGAGCAACACCGGTGGGAACTGCAATGAGATCACCCTCATCGAAACGGTGCACCTTCTGGTGACTATCCTGTTGTTGCTGTTGGCTTTGGTCTTCTCCTTCAAAACGTCTTGGTGCTCTTTGCGACTGATGCCGGCGACCTTGTTGTGCAGGCTCTTCATATGTGCTAGGACAACCAGGGAATATCAAACCAAAGTATCCCCTTCCTTGCTGGATGAAGATCTCCTGGGGAGCATTGGAGTAGAAAGGCCTCCGAAGGGCGTTGCGGCGGAGGACTAAGCGCGAGAGGGCGACGCCGGCGCATTCGAACTCCTGGTTGTTTGGGTTCCAAGTCTCAATGTAACCGCCCTCCGATTCAATGCGGTTGTCAGGCCTCTGCGCATTGAGGCGCTGGAACTGGCACGCATTTTCCTCCGGCTGCTGCCTGAAGGAGATGCTGCTAGCTCCCAGAACTAGAAAGCAAAAGCAAAAAGAAAGCGCCAGAAGCTTCGCCATGGCTGCTATTATTGTTATTGCT

EST-26

>SunSeed_CL851Contig1

TCCTCCAGGGATGTAAATAGTAACGCACAGAAACCTTGCTCAGTACTTGTAGTCCTTAACATGAAGGCTCTCTGAGGCACCCTCACCAAGGTTTGCACTACCCTTGTATGTTCCGAGAGTTGCTTCAGAATTGGCCTTTGCCCTTGTGAGGAATGCTTCTTGAGCCTTCTTCACATTCTCCTCTTTTCCACTCCAAGCCTTAAGAGTGCTCTGTTGAAGTGCCCTTCCGAAAGAGAAGGAAAGGGACCACGGCTTCTTACCCTTGACTTGGTTTATGGCATTGAGGTTGAGGGTTGCCTCCTCCTCACTCTGTCCACCAGAGAGGAAAACCACGGC**A/C**GGGACAGCAGACGGGACAGTTCGCTGAAGAGCTCTCACAGTGTGCTCGGCAACAACCTCGGGGGCAACCTTTGCAGAGTCAGATCCGGGGGTAACCATGTTTGGTTTCAAGAGTGTTCCCTCAAGCAAGACATGGTGGTCATTCAGTGCCTTATAGCATGCTGCAAGCACTCGCTCTGTTACTGCAGCACACTTGTGGATATCATGGGGTCCATCAACAAGGATCTCAGGCTCAACAATGGGAACAAGACCATTCTCCTGGCAAATGGCGGCATATCTGGCCAATCCATATGCATTCTCGTGGATGGCAAGTTCGGATGGCTCGGTGGGACCAATCTTGAGCACAGCACGCCATTTGGCAAAACGCGCACCAGCTTCATAGTACTTGGCGCAACGCTGACCAAGGCCATCAAGACCCTGAGTGGTGGTTTCTCCATTGGTTCCGGCAAGCTCGACGGTACCCTTATCAACCTTGATACCTGGAAGTACACCACCTTCCTTCAAGACCTCAACAAAAGGTTTGCCTGCAGCAGTGCTCTGGTAGAGGGTTTCCTCAAAAAGGATAACTCCACTGAGATACTGAAGAGCTCCAGGAGTGGTAAAGAGGAGCTCCCTGAGAGACCGCCTGTTAGTTTCGACGTTCTCGACATTGATGCTGGATAGACGCTTGCCAATTGTTCCAGTTGACTCATCAGCAGCAAGAATTCCCTTTCCGGGGGTGCCAATGTATGCAGCATTGGCAATCAGCTCATCATGGTACTTGCTCTTGAAGTTCGACATGGTTGATTAGATCGGAGAACGGAAAGAGCGATAGAGAGAGGGTTAGATAGTGAACGATGATAACGGACTAAGAAGGAATAAAAGAGAAGTAGAGAGAAAAAGGCGAGGGTTTTAGGGTTTTTATAATGAAA

EST-33

>SunSeed_CL14Contig1

AACCAAACATAGTAACAAAACACTACCAACAATAATCACAACACCATGGCCAAGCAAACCCTCTCTCTCTTTTCCTCCATAGCCATCTTCCTCGCGCTACTAAACCTCGCAACCTCACAAGATTCACTTTCCTTCAGCTTCAATAACTTCGAACAAGAAGCTGAAAAAAACCTAATCTTGCAAGGAGATGCACACATTGACCCAAATGTACTGCAACTCACTAGAACCGACAGCCAGGAAATCAGTGTTGGGAGAGCCTTGTACTTAGCACAAGTGCACCTATCGGATAAAAGTACAAACAGACTCGCAAACCTTCAAATCCAATTCAGTTTTTCCCTTAAGTCACGGGGCTCCTCCCACCCGGCGGACGGCCTTGCCGTCTTCCTGGCGCCGGCCGACACCACCATACCGCCCGGTTCAAACGGAGGGCTTTTAGGGCTCTTTGAACCTGACAATGCCCTAAACGCCTCCGCAAACAAAGTCATCGCAGTCGAATTCGACACCTTTTTTGACAGAAGTTCAAAT**T/G**CCTGGGATCCAAGTTACACACACATTGGAATCGATGTGAATTCCATTAAGTCTGCGAAGACTGTGAGATGGGATAGAAGAGACGATCAAATCCTTAATGTTCTTGTGACATACACTGCTTCTAGTCGAACCCTGGCAGTGTCTGCTAACTACCCTGATGGTAAAAAGTACGAGCTCTCTCATGAGGTCGACTTAGCCAAAGAGCTTTCGGAATATGTTAGGGTTGGATTCTCAGCCGCTACTGGACAACGATTCCAATCACACACTCTTCACTCGTGGTCATTCACTTCAGTGTTGCTTAATACTGTGACCATGGAGAATGAGTACCTTGTGTTTACACGAGGAATTGAGAAATAATGATGTGATATGATGATCATGTCTAATAAAAATATCAATCACATATGGCCATGTAAGATATGCCAATAAAGGACAAAAGTTGTACGTCCATGGCTTATGTATGTTGTGGTTTTTGTTGCTTATGTTTAAGCTAAGCCAATCATGTGATATAAAATGAACTACTACTTTGTATGAATGAGAGTATGTATAAAAAAAAAAAAAAAAAAAAAAAAAAAAAAAAAGAAAAAAAAAAAAAAAAAAAAAAAAAAAAAA

EST-45

>SunSeed_CL100Contig2

TAACCTTCCCCTACCATTATATCTATACTAACAATTAACGACAACAAAACATTAAAAAAAAACGCCAGAAAAATATTTAACTATCCCTAACAAACCAAGCCACTTTTTCACTTTCACTTCTTAGTCTTCTTCGCCGCTGCTTTCTTCGCTGGAGACTTCACGCTTTTCGGCTTTTTAACTGGCGTCTTCTTCACCGGCTTGGCAGCAACCTTCTTCGCCGGAGCCTTCGCTTTTG**T/G**CTTCCGCTTCGGCTTTGCAGAAGTAGGGGCAGTTTTGGCTTTAGGTTTGGCAGCGGCCTTCTTCGTAATGGCAGCTGGTTTGGCTTTAGGTTTGGATGCAGGCTTGGGACCAGGCTTGGAAGCCGCCGCCGCTTTAGGCTTGGCGGCGGCAACAGAAGAAGCCTTCGCTGGAGCCTTCACTGGAGCGGGAGGAGAAGCAGCTTTCGGCGCAAGCTTGAATGAATTCTTCACTTTCACGAGCTTTCCAGCAGCGACGGATTTCTTGAGGTTGTGGAGAAGAAGCTTCCGGAAGGTTGGTGGAAGATTCTTGTGCTTCTCTTCGATGAACTTCGTTATCGCATATTGACTTGAACCAGTTCTCTCTTTCAAACTCGAAATCGCGTCCGTTATCATCTCGGCGAAGGTGGGGTGAGAAGGAAGCTTCTTGGACGAAGCCGTTTTCTTGGTCTTGGGCTGTGCTGCGGCGGTAGACATGGCTGAAACTGAACCACAAAATTTAAACCAGAGAGGTTGCAATAACCGTTTCCGAGAGAGAGAGAGAGAGAGAGAGAGAGAGAGTGCTTTATGTGATGAAGA

EST-48

>SunSeed_CL112Contig1

AGTGTTCCTGTATATTTAACGTAGTGGAAGAAAACATAAGAGAGTGTTATTTGCGAGAAAAAAAGAAAAGAGAAGCAGAAAAATGGGGTCTGAAGCTGCCATACACGTCTTGCTGATTTCGTTTCCAGCACAGGGACACATAAACCCTCTCCTAAGGCTAGGAAAGTGCTTGGCCGCCAGGGGCTTATTCGTCACTTTCTCCACCACCGAGGATGCTGGCAAGGACATCAGAAACGCCAACAACATCGCCGAGAAGTTCGTTGCTCCGGTTGGCGATGGTTTCCTCAAGTTCGAGTTCTTCGACGATGGCTTACAGGACGACGATCCCATCAGGAAGAATCTGGCAGATCACACGAAGCATCTGGAAGTTGTCGGCAAGAGATTCGTTTCTCAAATGATCAAGAAGCACGCAGAGTCAAACCAACCAATCTCTTGCATCATAAACAACCCTTTCTTCCCGTGGGTTTGCGACGTTGCTC**T/A**AGAACACGGTGTTCCTTCTGCTCTCTT**G/A**TGGATTCAGTCAACGGCAGTCTTCACCGCTTACTATCACTACCACCACAAACTGGTTAGCTTCCCTTCACACGATCAACCTTACATCGATGTTAATTTACCTTCCGTTAC**G/T**CTCAAACACAACGAGATCCCAGATTTCTTGCACCCTTTCAGTCCCTTCCCGATTCTGGGAACAGTCATATTAGAACAGTTCAAGAACTTGTCCAAACCCTTCTGCATCTTGGTGGACACATACGAGGAGTTAGAGAAGGATTTCATCAGCTTCTTCTCAAAATCAATGAGCATAAGGCCCGTGGGCCCGTTGTTCAAGAACCCGAAAGCAAGAATGGGAGGAAGTAACATTCGTGGAGACATGATAACAAAGTCAGCGAACTGCGTTGAATGGCT

EST-66

>ALB-3-8C2-TriPEXPSP

AACCATAACACGGTAACAACTTTCAATTTTTCAGCCCCAACCAAAAATAAAGATAAAGAAAAAAAGGAAAACGCAACCTTCTGATTAGAAAGCATGGGTGTGGCCATTGAAGATGAAACGAAACCCAAGCCCATCGTCATCCGTGAGGTGTGGGCCTCCAATTTGGAATCCGAATTTGAACTCATCAGAGAAGTCATCGATAACTACCCCTTCATTTCCATGGACACGG**A/G**TTCCCCGGAGTAATCTTCCGTCCGCAGCCTACAGATCCGTCCAACCCTTACCTCGGGCGACGCCTCAGCCCCTCCGATCACTACCTCCTCCTCAAGTCCAACGTCGACGCACTCAACCTCATCCAAGTTGGACTCACTCTCTCCGACGAGAACGGCAACCTACCGGACCTCGGCACCGGTAACCGCTATATCTGGGAGTTCAACTTCAGCGACTTTGATCTGGCGCGTGACGCACACGCTCCTGACTCAATCGACCTGCTCCGCTGCCAGGGCATCGATTTCCGGCGCAACGCCGAAGACGGTGTGGAGTCAAGGCGGTTCGCGGAGCTGATGATGTCGTCGGGGATGGTGTGCAACGACGCCGTGAGCTGGGTAACGTTCCACAGCGCTTACGATTTCGGGTACTTGGTTAAGATCTTGACCCGAAGGGCGTTACCAAACGGGTTGGAAGAGTTCTTACGGGTTCTGAGGGTTTTCTTCGGTGATAACGTTTACGATGTGAAACACATGATGAGGTACTGTAAGGCCCTCTATGGCGGTTTGGATCGGGTCGCCCGAACCTTGAATGTGGATCGGGCCGTCGGTAAGTGCCACCAGGCCGGCTCTGA

EST-70

>ALB-3-8E_7-TriPEXPSP

CCCAAAAGCAAATTAAGCTATTAGAAAATCTCAACAACCATGGCTACTCTCTCCCCTCTCTCTTTCTTGGCCACAACCAAGGTCTCTGCATCCTCGCCATCCTTGCTTCCATCCTCCTCCAGAAAACCCTCCAAAGCCAGCCACCGAATTGCAAAGGTGTCATGCAATGCTAGCAACCCTGAAGGAGAACCACAAAACAACAGGAGGGATGTTCTGGTTGGCCTGGGAGGACTGGGGGCGGCGGCCGCCACTTTCTCCTACAACCCTTTGGCCTTGGCGGATGCGGTGGCCACCGACGTACACGCTTGCAACAAGCCAAGCCTTCCCCCAGGCGCAAAGCCCATCGCGTGTTGCCCTCCAAACTACGAAAACAAAGAACCCATTGAGTATGTTATCCCAAAGTACACAAGCCTAAGGATTAGAACACCTGCTCATGATTACGCTGACTTAGACAAGTACACGAAAGCCATTACCATCATGAAATCTCTTCCAGACGATCACCCTCACAGTTGGACTCAACAGTCTAGGATCCACTGTGCCTATTGCCACAACTCCTATACCCAAGTGGGTCACAAAGGCGTTGAAATGCAGGTCCACTTTTCTTGGATCTTTTTGCCTTTCCGTCGCTGGTACCTTTACTTCTATGAGAGAATCTTGGGGAAGTTGATCAA**T/C**GATCCCACTTTTGCATTGCCCTTCTGGAACTGGGATGCCCCTGAAGGCATGGAGATGCCTGCAATATTCACAGACAGGAGATCCCCACTCTACAATGCTCTGAGGAACCAGAACCATCTACCACCAACGCTCGTAGACTTGAACTGGAATGGTAGGAGCACGGGTGTTGATGGGAGTGGTGACGTGGAAGCCAACCTGGCAACAATGTACAGACAAGTGTAC

EST-80

>ALB-3-26G3-TriPEXPSP

TCATCGTTCTAGTTACTTGTTTTGTTGCAATTTTCGCTGGTGTTTCCGATTCTTAAACTGAAAATGTCGTCTTGCTGTGGAGGAAACTGTGGGTGCGGAAGCGGCTGCAAGTGCGGCAACGGCTGTGGAGGTTGCAAGATGTACCCAGATTTGAGCTACACCGAGAGCAGCAGCACAACAGAGTCATTGGTGATGGGAGTTGCGCCTGCCAAGGCCCAATTCGAGGGTGCTGAAATGGGTGTTCCAGCTGAGAACGATGCCTGTAAGTGTGG**A/G**CCAAACTGCAGCTGCAACCCATGCACCTGCAAGTGAAGAGCCCACACGCTCACACGCACAGCAGAAGCAGAGATCCTTTTAGTTAAATAATAATAATAATAATCATAATAATGATATCTAGTAGTGTTATTATGTGTATGTGTTACTGGTTTTGTGTACACTCTTCTGGAGGAGTGTTTAAAGAAAAAACACTCCCCGTTTTGGAGTGCTTAGAATAAAGTGGCCTGTGTCTTGCTGCATCCTTGAACTTTGGCTTGGGTGTATCTAGCCATGGGTGCTTTGTTTCTTTGTTGGTTTCTTTGCTTTTTGTATAAACTTCCGCTAATCCTGAATCGTCCATCTCTGCTGGTTAATGAAATGATATTAAAGAATTATCCAAAAAAAAAAAAAAAAAAAAAAAAAAAAAA

EST-87

>ALB-3-26H9-TriPEXPSP

CACACAACTAACACACTACTCCTCAAGCATCATCACATTCCACCACCACCATGGCCAAGTCCACCATCCTGGTAGCCCTCCTTGCCCTCGTCCTGGTGGCACACGCCTCCGCGATGAGGCGCGAGAGGGGGCGACAAGGGGACTCATCAAGCTGCGAGAGGCAGGTAGACAGGGTGAACCTCAAGCCCTGCGAGCAGCACATAATGCAGAGGATAATGGGCGAGCAAGAGCA**G/A**TACGACTCCTACGATATTAGGAGTACTCGATCCTCCGACCAGCAACAGAGGTGCTGCGATGAGCTGGACCAGATGGAGAACACGGAGAGATGCATGTGCGAGGCATTGCAGCAGATAATGGAGAACCAGTGCGATAGGTTGCAGGACA**G/A**GCAAATGGTGCAGCAGTTCAAGAGAGAGCTCATGAACTTGCCTCAACAGTGTAACTTCAGGGCACCACAGCGTTGCGATTTGGACGTGAGTGGCGGCAGATGCTAGACTCAAAAATAATAATCTGTGCCAAAAGAAACTAGTAGGAAGTAGCTTATGAGCTATTATGTATGCTTGTTTCGTTAATAATAAATATCATCACTGTATGAATGTGGTGATAGGTAAGGTTATATGAGCACCTTCGGTGTGCTCTTATGGCTTTACCTATGTTTTGCTACTGCA

EST-92

>ALB-3-27B4-TriPEXPSP

CTCTCTTTATTAGGGTTACGGTCGTTCTCTTTATTTGGCTCTTTACCTCTCGATTCCCTTTGTTCTTCTCTGGTTCCATTCCCCGAGATCTGCATCTCTAGTTGCAGGAGTAATCGTTGAGAAACTCGCGAACTGAAAAAAATGGCGGAATCATCTCGCGAGGAGAACGTGTACATGGCAAAGCTTGCGG**A/T**GCAGGCGGAGCGATACGAGGAGATGGTGGAGTTCATGGAGAAGGTGGCGAAGTCTTCTGACAACGAAGAGCTGACGGTGGAGGAGAGGAACCTGCTGTCAGTTGCTTACAAGAACGTTATCGGTGCGAGGAGGGCTTCGTGGAGGATCATCTCGTCGATTGAGCAGAAGGAGGAGAGTAGGGGAAACGAGGACCATGTTGCAACCATCAAGGAGTATAGGGGAAAGATTGAAGCTGAGCTCAGCAAGATCTGTGATGGCATTTTGAACCTCCTTGAGTCCAACCTTGTGCCCTCTGCTACCACCCCTGAGTCCAAAGTGTTCTATCTCAAGATGAAGGGTGATTATCATAGGTACCTCGCTGAGTTCAAGACTGCTGCAGAGAGAAAAGAAGCTGCTGAGAGTACTTTGTTAGCGTACAAGTCTGCTCAGGATATTGCTCTTGCAGACCTTCCTCCTACTCACCC**G/T**ATAAGGCTGGGTCTGGCACTCAATTTCTCTGTGTTCTATTATGAAATTCTTAACTCACCAGATCGTGCTTGTAATCTCGCCAAGCAGGCATTTGATGAAGCAATTGCTGAGCTTGACACCTTGGGTGAAGAATCATACAAGGACAGTACCTTGATCATGCAACCTCTCAGAGACAATCTGACTTTGTGGACATCTGATATCACGGA
