# Supplementary material for: Identification and Evaluation of Single-Nucleotide Polymorphisms in Allotetraploid Peanut (Arachis hypogaea L.) Based on Amplicon Sequencing Combined with High Resolution Melting (HRM) Analysis
Source: Front Plant Sci. 2015 Dec 2;6:1068. doi: 10.3389/fpls.2015.01068 (PMC4667090; doi:10.3389/fpls.2015.01068)
Supplement: Supplementary file 4 [file DataSheet4.DOC]

**Additional File 4: List of genomic-SNPs identified in *A. hypogaea***

Description: – Forty four genomic-SNPs were identified and twenty two were used for genotyping. SNP used for genotyping were shaded in yellow.

GSS-2

>AdH1011-CPY2-J24TF

TTTTCAGTTTCCCTCCAATCCTTCTTATGACTTAAGATCTCTTTCATGAACTTAGCATAAGAGGGTATTTGCTCAAGTGCTTCTGCAAACGGAATCTTTATTTCAAGAGTCCTGAGATAGTCTGCAAA**G/A**CGAGCAAATTGCTTATCCTGTTCCGCTTGGCGGAGTTTTTGAGGATAAGGCATTTTGGCTTTGTATTCCTCAACCTTAGTTGCTACAGGTTTATTACCTACAGAAGTGGGTTGGGAAGCCTTTTTAGAAGGGTTGTTATCAGCACTTGTATGTGACTGATTCCCCACTGGTATTTGAATGCCAGTGGTGGGAGCTGGAGTGGCGTTAGACGCCCCTTCCTTGTTTGTTACTGGCGTTTGAACGCCAGAACCATGTTCCCCTTGGGCGTTCAACGCCGGATTCATGCTTGTTTCTGGCGTTGAACGCCAGGAATGAGCATGGTCTGGGCGTTCAGCGCCA**G/TC/A**TTTGTCCCTTTCTGGGCTCGGACTGTCTTCAGGAGGATTTTGAGTATCCACTTGTTTATTTCTTGGTTTCCTGCTGCTTTGAAGTGAGGTATTTAATGTTTTCCCACTTCTTAATTGAACTGCTTGACATTCTTCTGTTATTTGTCTTGATAGTTGTTTTTCTGT

GSS-4

>AdH1011-CPY2-L12

CAACAAGATCACCATTGTCACTTGATGTAGAGTTATTTTCGTGGATGGAACTTCCTTCTTGTTCAACCTCCTCTAGGTCTTCTTCCACCTCTTTATCTTCAACAATCTCCATTTCCTCCGTTTGTTGCAACACACACTCCATTTCCTTATGCTCCACTTGAGAGTCTAAAGTCTCCCTCATACCTCCCTCTTTGGTTGCTTTCTCACAATCATCTGTGAACTCATTTCGGTTGTGATATGAATCCCAAGAATCTATCTTTTGACGGATGGTTGCTTGAAGTCA**G/A**TCCATTTCTTCCTTGAAACGATCCGTTAATTCTTGTTCCGCTTTATTAACATAAGTAGGATCATATTGCTCTTGGATTAATGGACATTGATATGCTTCCATGGAGGTTTGTGGCGGAGTGGGGG**A/G**TTCATGATATGGTTGGAAAGAAGATTCATCAAAGCTTTGAGGTGGGAAGTTATTTTGGTTAATGGTGGAAGGTGGAATTATACGGGATGTAAATTCTTGGAGGGTAGAGGTGAAACTAGTGAAGGCGGTTTGGAGTTCTTCTTGCTCTTGAAGAATGATACCAAGTGTATCGTTTATGGGGACTTGGGTTGGAGGGTAAAAATGTTGGAGTGGTGGTGATTCAAGGGTTGCTTGTTCATAATCGTCACAAGGGTATGCATAGGGGGAATATAGATTTGGATCATATGTAGGCGATTGGTGGAAATAAGATGTGGGTTGAGAATATGGTGCATAGAATGGTGGTGGTTGAGAGGTATAACCATGATAAGAATCATCATATCCATAGGAATGATATGCATTATAGGAGGGACTACCATCATAGTAACTTGAGGGGGGAGATTGCCATG

GSS-10

>AdH1030-CPY2-B13TF

ATGAGATCTTTAAGCTTCTCAGGTAAGCTTTTCAAAATGATTGTACTGCATTCTTCAGTGAGGTAAACTTTTCCAGTCTCCCTCCAATCCTTCTTATGACTTAAGATCTCTTTCATGAACTTAGCATAGGAAGGTATTTGCTCAAGTGCTTCTGCAAACGGAATCTTTATTTCAAGAGTCCTGAGATAGTCTGCAAAGCGAGCAAATTGCTTATCCTGTTCCGCTTGGCGGAGTTTTTGAGGATAAGGCGTTTTGGCTTTATATTCCTCAACCTTAGTTGCTGCAGGTTTATTACCTACAGAAGTGGGTTGGGAAGCCTTTTTAGGAAGGTTGTTATCAGCACTAGTATGTGACTGATCCCCCACTGGCATTTGAATGCCAGGGGTGGAAGCTGGAGTGGCGTTAGACGCCACCTCCTTATTTGTTACTGGCGTCTGAACGCCAGAACTATGCTTCCCTTGGGCGTTCAACGTCGGATTCATGCTTGTTTCTGGCGTTGAACGCCAGAAATGACCATGGTCTGGGCGTTCAGCGC**C/A**AACCTTATTCCTCTCTGGGCTCTGATTGTCCTCAGAGGGATTTTGAGTAGCCATTTGTTCATTTCTTGGCTTCCTGCTGCTTTGAAGTGAGGTATTTAATGTTTTTCCACTTCTTAATTGAACTGCTTGGCATTCTTCTGCTATTTGTTTTGACAGTTGCCT

GSS-26

>AdH1177-CPY2-P1TF

ATACTCAAAATATGTATCTTACTCTCGCATTTCTGTGGGACTATAGAATCCCAACATTCACCAATCAATCACACATTTTACATATTGTAACTTTATTTTAATTTTCATTGATAAGTGTCCTTACAAATCCTGCATGTACAGGCTTATTCTCTCATCCTCATACCTTAACATTTGAA**T/C**AAATTCAGGAGCTTCTGCAAT**G/T**GTGTCATCTAGAAAGCTCAATGTAGAATTACAGGACTCAGCGCAGTGCCAACTGTGATGATCTGCTGGAACAGAAGAGGCTTGAATAGCCTCACCCTTTTCGT**C/A**ACCATTCATTAGCTTGGAGAAGAGTGCTTTTGCAGCTTGGTTGCCAGAGAAGGAATTGGCCATTTTGTTGAGTAAGCATGGCACATGAAGCTGCTGCTGCTGGTGATGCGGAGGAGTGTGTAATAAGTGGTTGTTCTTGTTGAGAGAAGAGTTCCAGTAGTTCTTGATCTCGTTGTCAGTTCGATTAGGCAAGTGCTTGGCAATAGCGGACCACCTGTTGCCAAGAAGTGCATGGAGTCTTAGGATGGTCTGCTCCTCATCTCTGCTCAATCGGCCTCTCTTAATGCCAGGTCTGAGGTAGTTAAACCACCTCAGTCTGCAACTCTTTCCACATCTTT

GSS-35

>AdH1184-CPY2-J10TF

AATGCGGAGCCTCCATGGGAACATAGAGTTGAAGAAAACCCCTCCAAGATGATTGAAATTGATACTAGGGAGGAAAGTACACACCTTCCAAGGCATATTCCATATGAAGACTTGAATCGGATAGAGAAAGAATTGAGTTCCCTTGGTGATGAAGATCAAGCATCAAGTCTTAGTGGTGAAGAATCCTTTGAGCATGAAGAACCTTCTCTGGTTGGATTTGAAAGCGTTGAGGAGGTAAATTTTTCTCACCCTCCCTATTATGATTTGAGTAAACGAAAAGGTTTAGATAAAATTATTGAACAAAGGATTGAGATTAAGAGATCTTGTGAAGAGGTGGAAGTCCCTAAAAATAGAAGAACGGGGGTTGGTTA**T/A**GCTTTGTTAAGGTCTTTGGAAGCATCTTTGCCTAGGTTGCCATCTACACCTTCATTTGAGTGGGTGAAATTCATTTCTATTAGCTTTATTATCCCACTTGAATATGGTTTGCTTGAAACGGATTGCCAACTTAGGGAAGTTTGTGGGATGAAGCGTAAGCAAAAAAGGTTTTGTGGTGGGCGTTGCAAATCAAGGCTCATTATGGTTGATGCATCAAACATGAGATATAAAGGTTTGAGTAGTGCTCAAATAGATGGGTCTAGGAGGATTGTTGGCCACTATATAGAGAATTCACCCTGCTCACCACCCGGTTGGACTAATAATGATGATCAACCTCAAGACGGGTGTGAAAACAAAGTGTGGGATCCCGGATTACAAGAAGAGAATCAACTTTGGGAGCCCCAAGCTTGTGAAGAACTCCATCAAGACTTGGCTCAA

GSS-44

>AdH1184-CPY2-N2TF

AACTTGTACCAAGCAAGCTTTATGTGATCTTGGGGCAAGTATCAACCTAATACCTGCATCTACTATCAGAAAGCTTGGTTTGACTGAAGAAATCAAACCAACCAGGATATGTCTTCAACTTACTGATGGCTCCATTAAATACCCATCAGGCATGATTGAAGACATGATTGTCAAGGATGGGCCATTTGCCTTTCCTACTGACTTTGTGGTGCTGGAAATGGAGGAGCACAAGAGTGCAACTCTCATTCTTGGAAGACCTTTCCTAGCAACTGGCCGAACCCTCATTGACGTCCAAAAAGGGAAAGTAACCTTGAGAGTCAATGAGGAGGAGTTCAAGTTGAATGTTGTCAAAGCCATGCAACATCCAGACACCCCAAATGACTGCATGAGTGTTGATATTATTGA**C/A**TCTCTGGTAAGAGAGGTCAATATGGCTGAGAGTCTCGAATCAGAGCTAGAGGACATCTTTAAAGATGTTCAGCCTGACCTGGAGGAATCAGAGAGAATAGTAGAACCTCTGAAAATCCCTCAGGAAGAGGAGAAACCTCCTATACCCGAGCTCAAACCATTACTACCATCCCTAAAATATGCATTCCTGGGAGAAGGTGATACCTTTCATGTAATCATAAGCTCTACCTTAGAGCCACAGGAAGAGGAAGCACTAATTCAAGTGCTAAGGACACACAAGACAGCTCTTGGGTGGTCCATCAGTGATCTCAAAGGGCATTAGCCCAGCCAGATGCATGCACAAGATCCTATTGGAAC

GSS-45

>AdH1184-CPY2-N6TF

ACTCGGACGCCTCCTTTGGATACAAGTACCTCGCTTTCATGGATGCTTATTCGGGATATAATCAGATTCTAATGTATCCACCTAATCAAGAAAAACCCTCATTCCTAACCCCAAAAGCAAATTACTATTATGTGGTCATGCCATTTGGACTCAAAAATGTAGGAGCTACCTACCAAAGATTAATGAACAAAGTCTATGCAGACCATATTGGGAAACTCAT**T/G**GAGGTATATGTGGATGACATGTTGGTAAAAACTCAAAGCGAAGAGTTGTTATTGTTTGACCTCGCCCAAGTGTTTAACACCATAA**G/A**GAAG**C/T**AT**G/A**ACATGCGACTTAACTCTGCAAAGTATACCTTTTCAGTAGAAGCTGGCAAATTTCTA/GGGTTTCTTACTGACACAATGAGGAATTGAGGCGAACCTGGATAAATGCCGGGCCATACTTGACATGAAGAGTCTGACCTGTGACAAAGAGGTACAATAACTCAACGGGAGATGGTAGCCTTGTCCAGATTTCTAGCCAGATCGGCCATACGATCTCTCCCCTTCTACGCCACTTTAAGGAAGGAAAAGAGGTTTGAGTGGACAACA**A/G**AGTGTGAGCAGGCTTTCCCGGATTTTAAAAAGTTCTTGGGTCAGTCACGTATTCTAACTCGACCACAGGAAGGAGAACCACTCATATTATACCTCGTAGTAGAAAATCGGGTAATAGCATCAGCACTAGTCAGAGAAGAGGAAAGGGGACAACAACCCATCTACTTC

GSS-46

>AdH1184-CPY2-N8TF

ACATCACACATTAGTTCAAATGGTAATGTCCAGTCTGGTGCAGAGATGACTGGTGTTGTGACCAGCTTAGCTTTCAGGGTCTCAAACGCCTGCAGACACTCCTTATCAAAGATAAATGGCGTGTCAGCAGCTAGCAGATTACTCAGAAGTTTGGCAATTTTTGAAAAATCCTTTATAAACCTTCTGTAGAATCCTGCATGCCCCAGAAAGCTTCTGATTGCCTTAACATTGGCAGGTGGTGGTAATTTTTCAATTACCTCTACCTTTGCCTTATCCACCTCTATTCCCCTGCTTGAAATTTTATGCCCAAGGACGACTCCTTCAGTCACCATAAAGTGACATTTTT**C/T**CCAGTTTAAAACTAGGTTGGTCTCTTGGCATCTTTTCAGAACAAGTGCTAGATGGTTAAGACAGGAGCTGAATGAGTCTCCAAATACTGAAAAGTCATCCATGAAGACTTCCAGAAATTTCTCTACTATATCTGAGAAGATAGAGAGCATGCACCTCTGAAAGGTTGCAGGTGCATTGCACAGACCAAAAGGCATCCTTCTGTAGGCAAATACTCCAGAAGGACATGTAAATGCTGTTTTCTCTTGGTCCTGAGGATCTACTGCAATTTGGTTGTAACCTGAATAGCCATCCAAAAAGCAGTAGTATTCATGACCTGCTAGTCTCTCTAGCATCTGGTCTATGAATGGTAAAGGAAAATGATCCTTTCTGGTGCTGTATTGAGCCTTCTGTAGTCAATACACATGCGCCACCCTGTAACTGTTCTTGTAGGACCAGTTCATTCTTTT

GSS-49

>AdH1184-CPY2-P12TF

AGCATCAAGACCACTTCTATGAAGTTGTGGCCTTGAAGAAGGTGATCCCCGAGGTCCCTTTTTCACTCAAAAAGAGTGAATATCCGGAGATCCGACATGAGATCCGAAGAAGAGGTTGGGAAGTTCTTACCAACCCTATTCAACAAGTCAGAATCTTGATGGTTCAAGAGTTCTATGCCAATGCATGGATCACCAAGAACCATGACCAAAGTATGAACCCGGATCCAAAGAATTGGTTTACTATGGTTCGGGGGAAATACTTGGTTTTTAGTCTGGAAAATGTAAGGTTGGCATTCAACTTGCCCATGATGCAAGGAGATGAACACCCTTACACTAGAAGGGTCAACTTTGATCAAAGGTTGGACCAAGTCCTCACAGTCATATGTGAAGAGGGCGCCCAATGGAAGAGAGATTCAAGAGGGAAGCCGGTTCAATTGAGAAGGCATGACCTCAAGCCCGTGGCTAGAGGATGGTTGGAGTTTATTCAATGCTCAATCATTCCCACTAGCAACCGGTCCGAAGTT**A/C**CCATAGACCGGGCTATCATGATTCATAGCATCATGATTGGAGAAGAAATAGAAGTTCATGAGGTTATAGCTCAAGAACTCTATAAGGTGGCGGACAAGTCCTCTACCTTGGCAAGGTTAGCCTTTCCTCATCTCATTTGTCACCTCTGTTATTCAGTTGGAGTTGACATAGAGGGAGACATCCCTATTGATGAGGACAAGCCCATCACTAAGAAGAGGATGGAGCACACAAGAGACCCCACTCATCATGAGATCCCTGAGATTCCTCAAGGGATGCACTTTCCTCCACAAAACTATTG

GSS-52

>AdH1011-CPY2-I12TR

GCGAAATGTTCACACTAAGCTACACTAAACGAGGCACAAGATATACATACAGATATCAAGACATACATATAAACATATCAAAGTATAATAGTCATAAGGATCTAGCCGCCGCCAACGGAGTTTAAGCCGGCTAGTTATATATCGACATACAGAGTTTTGAAAGTAAAACAGCTTATACAAGTTTTTCTCTCAAAGTAAGCCTCTAGGAAAAATAAATACAAAAGTGAGAGATCTTAAACAAAATAATCAAAAAGACTTCAAAATATGATTGAGATCCTCCGCTCTGTCACCCCCAAGTAACTCACCAAGGAGGGTTGCGACCTGCATCTAAAAAACAACAACAAAGTATGGAATGAGAATCGGAGGTTTTCTGTATGGTAACAGTGCCCAATAATGTAAGATGTAAGGTTCCAGGACGCCAAATGCAATCCTAAAACTTCACCCAAATGCAGATATTCAAGCTTAGAATAAGAATAATTAAAGAACTTAAGCCTTAAACAGGGGTAGCTAAACTTAGGGGAATTCTAACTAATACTAATCACACCGTTGTATTTCGCAGCCTTCACCAACCTAACCTCCGTGCAATCCCATCG**C/T**CACCTCCTACCAAGCCTCCTCAATCCCAACAGAAGACACAGATAATGCATACAAGTAAAGCACAAGTAATATACATATACAGCAAGTAATCAATTAACAAGTATACATATTATACAATTAGGCAAACTCAAGTAATCAAATCAATCAAGCATATAAGAGATACATATAATGAATGACTGTCCTATTGTCT

GSS-54

>AdH1011-CPY2-I16TR

AAATGACTCATCAAAAAAATCAACATAAAAGAACTTGTAAGCAATAGGATTGGAAAGTCTAGGTTCAGCAACAGGACGAAGATGAAAGTCAATAACAGTACCGCGAGTATGGCGAGTCTGAGTGGGGGGATGCTCTTCGTCTTCGTGAAGATGCCTCTTTACTCTGGAAGAACTGGGTTTGGATGAACTGGGCTGAAATGTGTTTGGCGGAGGAGGATGAGAAGGGCAAGGAGCAACGCCAGGGCGGCGAGCAGTGGTCTTAGTACGGGCCATATCAGAAGAAGGGTTTTGTGATGATGGAGGTGAAGAATGAGGAGGTGATTGTGAGGGAGATGAGTGTGTGTGATCAGAACAGTGGGTGCCACTATGGGATCCTTTGCGGGCAGCTTGTTTCTTTCGTGCCATATTTATAGAGCACTGGTTTACAGAAGATGAAAGAATAAAGTGTGTAGTGTGAAATGATGCATGTAGTGGGAAGTTAATATAGGTGTGGAGGAGTGCAAAGGTTGCGTCTTGAAAACAAGTAAAAATAAAAACTTTAAAGTGGTTTTGAAAAACATTGAAAGTTACAGTTGTAACCTTCGTACGTTGATA**C/T**TGGGAGAGAGAAACCAAATACTTTAAAAACCTAATGAAAATGGAGCAACCAAAAAATTCGAAACCCATTAGATGATGAAAAAATGGAGGGGGCCCAATTAAGGTGAAATGCAGTTGTTAAAACATTGAGAAAGCAAAAACTGAAAAGGGCACAATGGTCTCAATAAAGAAAAGCCCATAGGTCCAGAGACTGCTTCAGCACTTGAAGTCCATCATTCAGATTTGAATTCAATAGCTTATAGAGGTACTCATCATCTGCCAAAGAAAATC

GSS-56

>AdH1011-CPY2-I20TR

GTAGAGATGACTGGTGCTGTGACCAGCTTAACTTTCAGAGTCTCAAATGCCTGTAGACACTCCTTATCAAAGATAAATGGCGTGCCAGCAACTAGCAGATTACTCAGAGGTTTGGCAATTTTTGAAAAATCTTTTATAAACCTCCTATAGAATCATGCATGCCCCAGAAAGCTTCTGATTGCTTTAACATTGGCAGGTGGTGGTAATTTTTCAATTACCTCAGCTTGATCCACCTCTATTCCCTTGTT**C/T**GAAATTTTGTGCCCAAGGACAATTCCTTCAGTCACCATAAAGTGACATTTCTCCCATTTTAAAACCAGGTTAGTCTCTTGGAACCTCTTTAGAACAAGTGCTAGATGGTCAAGACAAGAGCTGAATGAGTTTCCAAATACTGAAAAGTCATCCATGAAGACTTCCAGAAATTTTTCCACCATATCAGAGAAAATAGAGAGCATGCACCTTTGAAAGGTTGCAGGTGCATTGCACAAACCAAATGGCATCCTTCTGTATGCAAATACTCCGGATGGACATGTGAATGCTGTTTTCTCTTGATCTTGGGGATATACT**G/A**CAATTTGATTATAACCTGAATATCCATCCAGGAAGCAGTAGTATTCATGACCTGCTAGTCTTTCTAGCATCTGGTCTATGAATGGTAAAGGAAAATGATCATTTCTAGTAACTGTATTGAGCCTTCTGTAATCAATACACATACGCCACCCTGTAACTATTCTTGTAGGAACCAATTCATTTTTTTCATTATGAACCACTGTCA

GSS-67

>AdH1011-CPY2-M10TR

ATTAGTTTGTGGGTGATATGGAGTAGCCACCCTGTGGTTGACTCCATAACGTACTAGAGCAGAGTAGAGCTGTTTATTACAGAAATGAGTGCCCCCATCACTGATTAATACTCTAGGGATACCAAATCTGCTGAAGATATGTTTCTGGAGGAATTTTAACACTATTTTAGTGTCATTAGTGGGTGTTGCAATAGCCTCCACCCATTTGGATACATAATCCACTGCCACCAGAATATAGGTGTTTGAGTATGATGGCGGGAAAGGTCCCATGAAGTCAATGCCCCATACATCAAACAA**C/T**TCAATTTCCAAGATTCCTTGTTGAGGCATAGCATAACTGTGAGGCAGGTTGCCTGATCTCTGGCAACTATCACAGTTAAGCACAAATGCTCGGGAATCTTTATAGAGAGTAGGCCAGTAGAAGCCACTCTGGAGGACTCTTGTGGCTGTTC**G/T**TTCACTTCCAAAATGTCCTCCATACTGTGATCCATGGCAATGCCAAAGGATCC**T/C**CTGTGCTTCTTCTTTAGGCACACATCTACGGATTACTCCGTCTGCACATCTCTTAAAGAGATATGGTTCATCCCAAAGATAGTATTTTGCATCTGTGATTAATTT**C/T**TTTGATTGCACCTTACTGTACTCTTTGGGTATGAATCTCACTGCCTTGTAGTTTGCAATGTCTGCAAACCATGGCACTTCCTGGACGGCTAGTAATTGCTCATCCGGAAAGGTTTCAGAAATTTCAGTGAGAGGGAGGGACGCCCCTT

GSS-69

>AdH1011-CPY2-M14TR

GATCCCCGAGGTCCCTTTTTCACTCAAAAAGGGTGAATATCCGGAGATCCGACATGACATCCGAAGAAGAGGTTGGGAAGTTCTTACCAACCCCATTCAACAAGTCGGAATCTTGATGGTTCAAGAGTTCTATGCTAATGCATGGATCACCAAGAACCATGATCAAAGTGTGAACCCGGACCCAAAGAATTGGCTTACTATGGTTCGGGGGAAATACTTGGATTTTAGTCCGGAAAGTGTAAGGTTGGCATTCAATTTGCCCATGATGCAAGGAGATGAACACCCTTACACTAAAAGGGTCAACTTTGATCAAAGGTTGGACCAAGTCCTCACAGTCATATGTGAAGAGGGCGCCCAATGGAAGAGAGATTCAAGAGGGAAGCCGGTTCAATTGAGAAGGCATGACCTCAAGCCCGTGGCTAGAGGATGGTTGGAGTTTATTCAACGCTCAATC**A/C**TTCCCACTAACAACCGGTCCGATGTTACCATAGACCGGGCTATCATGATTCATAGCATCATGATTGAAGAAGAAATAGAAGTTCATGAGGTTATAGCTCAAGAACTCTATGAGGTGGCGGACAAGTCCTCTACCTTGGCAAGGTTAGCCTTTCCTCATCTCATTTGTCACCTCTGTTATTCAGTTGGAGTTGA

GSS-72

>AdH1011-CPY2-O6TR

AGGTGTCCTTCTGGCATGCTTCCAGAATGGAGCATCATATGTATATTCTATGATAGTCTATCTAAGTTGTCAAAAATGTCATTGAATCATTCTACTGGTGGGTCTGTTCATCTGAAAACCCCTGCAGAAGCCCAGGAACACATTGAGATGGTTGCAAATA**A/T**CCAGTTCATGTACACTTCTGAAAGAAATCTGTGAATAATAGGATGACTTAGAAGAAAGAGTTCTTGAGGTTGATACTCTGAATGCTATATTGGCTCAGAACAAAATATTGACTCAGCAAGTCAATATGATTTTTCAGACTCTGATTGGATTGCAAGCTGCATCTGGCAGTACTAAAGAAGCCTTGACGAGTGGATTTCTATCGGTAAAGAAATTCACAAATATAATCGCGTTGTAAGTATAGTTTCTAAACCAACAGAAAATCCTTTCGTGCAAAAGTTTTAGTTGTCACAAGTAACAAACCCAGTAAAATTTATAAACCGAAGTATTCAAACCTCGGGTTGTCTTCTCAAGGAATTGCAGGGAGGTGTGTTTTATTATTGGTTATGGAAAATAGTATTTTTGGGTTTTTGAAAGGTTTGAACAAGAGAAATAAATTGCAGGAATTAATAAATCAATGGCTAAGAAAACTCTTGGCAAGGTATGCAAATTAGAAGTCATATCCTAGTTATCCTTATCAATTGTGATGAGAATTACTCGTTGCTCCC

GSS-74

>AdH1011-CPY2-O12TR

TGATTCGCTTAGGATGTAGGTATGATGTCCTTGTGAGCATGGCAATCTACTTGATCAAATTCATACTAAAATCCCAGAGTTTCTTAGCCAAATCAGCATCAGTTCCATGGGAGGTTGTTTTGGACAGATTGCTATCAGAGAAGTATTTGCCACTAACTCCCTTCACTTGTGGGTGCAATGCTACATAGCATGTTGTTGCTGCTCCCTACATAGCATG**C/T**AAAATCTCATATTCAACATGATGAATGTTTCAACTTTCAACTATCTTGTATGCTAAATTACTCATGTGTCATACCTGCTGTACATTTTTAAGCACAAGTCTCCCAATTGCATTAACTATACCTGCATGAACCAAAAAATTGGATGGAAAATGAACCAAAAGCATACTTTATGAGTTCATAAAATAGTAGAATGTGGTAGTAGTTACCATTGGCCACACCCATATGACGGAAAAGGTTGGTGGCAATGGTTCCCGGATGAAGAGAATTCGCAGTAATATCAGCCCCGTCTTCCTAATCATGATTCAGGAAGCAAGAATTAACATTAAAGAGTGACGGCATAAGAGAGTAAAAAAGCAACTAACCTTAAGAAGTGTTGTGAGTTGATTAGCATGTAAGATGTTTGCTAGCTTGGACTGCCCATAAGCACGCCAGCTGTGGTAACTATAAAATACAAGGAAGCAAAATTAATTACTCATAGAACAAGCTAAGTATGAATTTGAATATGGATATATACCTTGATTCGTCGTTGATTTTGTCAAAACGAATTCCTTCAGAATAAGTAAAACGGTGAGCC

GSS-76

>AdH1155-CPY2-I11TR

CCCTCTTTGGTTTCTCCAACTATGGCTCAATGCCATTTTTGAAAAGTATATGATAAAGCCTAGAGTTGGTGCTACAGACAAACAGCACATCGAAGAGTTCCGATTGGCCAGTTTCAAGCCAAATTTCCCAGAAGCCCAATCGGATGAAAACTGATTTTGGGCTGTATTTTCTCTTTTTCATTCTTGCAAGGACTTCCATAATGAGAATCTCAATTTCACCCCTTTTTTGCATCGAAATTGTGGTCCGGCTTGGCTTGAGCGCCTCTTCTTCCCAACAATATTGAAGAAAATGAGCTTGCTAACAGGAGTTGGGCAAATTTGTTGGCTGTACAGGTAATTCCTACAGGGCTACCTCAACATAAAAAAGAACGTTTCAAAGTAACTCTGTATGCCCCCTCGTTACACAGCCATACAACCTAACTTCTCACAAGCTATCCCAACTCCACTCCCCAGAAACAACAGGCCATTTTGCCATATTACCTTAGCTTCGAAGAAA**G/A**AACTTGATCTCTGCCTCTTAAAAAATCAACAACAAAAAGAGGGTTATAATCACCTTGTTTACGATCGAAGCTCTTACATCACAAAATCCTACTTTGATTGGTGGACTGCTTACTACTCTAAGTACAGCCGCATTCTGGAAGAGATTAAGCATTCTGTTGTCCAAGTAGTAGGGGTGGCGAAACAGGCCGAACCCACCGGGTCAAACCACCAAAAACGGAGGATCGGGACGAGATTTTGAACCCGCCAAATTTTAAAAACCCGCCAAACCCGCACTGCCAAAATCGCGGGCCAAGGCGGTGCGGGGCGGGCTGGCCCGGCGGGCTGACA

GSS-80

>AdH1155-CPY2-I23TR

AAGATGAGTTCAAGCACAAGCTGCCATGATAAGGAGCTCCCCAAAATGTCCAACTTAAGGACAATAACTAAAAGTGCTAGGTGGGAGACAACCCACCATGGTATGATCGTTCCTTTTCAATTTTAATTTTGTTTCGTTTTGTTTGTTTTAAAGTTTTGTTTTATTATATTGAACCTAGAATTATTCATAACCTCCATATTAGCATTACATTCTGCATACT**G/A**CATTATTATATAAAAAAAGCATGTGACGTGTAAGCGTCGCTGACGCGTCCGCGTCATAGGTGCATAAGCGAAGAAAGGAAATTGAACAGAAAGTCACGCGGGAGCGTGGCTGGATGCGTGCCTTTGGCACCAATGCATCCACGCGGACGCATCCCTTA**T/C**GTGTCCGCTTCATTTG**T/A**GAAAATAGCTCTCCAC**G/A**CGCACGCGTGACCAAAAAAAT**C/T**GACATCAATAGTGTTTAGTCAGAGAGTTGAGCTGCCCTTGAG**C/T**T**G/A**GAATTGCG**C/T**TGCAGGTACAAAGTGACCCATGCGTATGTGTCCCTGACGCGCGCGCGTCCCATGGCTATTTCAGCTTTTATGCGTCTGCGTGATGCACGCGTACGCGTCGTGGCCCATTTCTACATTCTTACTTCTTTCTTCTCTCCTTTCTACTTCTTTCTTCCTTCTTTCTTACTTTCTTCTTCTTCATTCATTTAACCTCTCACCCTTCTTCAC

GSS-81

>AdH1155-CPY2-K1TR

CATTCTTCCCTTTTTCTTTCAGGATGGCCACCCGGAAGGGAACCAGAAGACGTTTACATGGGGAGACAGACAAGTCCATCTACAAAATCTTCGGACAAAAGTGCCAGTTGGAACAGCCCGTCCACCTGCACATCTCAGCATGCACCGAGGACGGTGCAATCTTTAAGTGTGGGGAGGTCGATACC**G/A**ATC**T/C**CCATGGGTTAGTTATT**C/GT/C**CTTCTCAACATCAATGTCTTATTTTCTTTATTAGTTCATTTTTGCATTGCATAATAGATTGCATGTGTAGTTGATTGTTTGCATTTACGTACTACTTGGTTGAAAAATAATAAGTCTTTCTAGGACCCTATTTTTGAAAAATTTCACTAATTTAAAATCAAAATTTTTGTGTTAAACTTGTTTGAAGTTGTATTTGGAACATGGTTTTTGAGCCAAAGAACACACAACCCGTGAAATTTTGAGCTTATTTATATGGTTACATCATTTGACCATAAATTTTTATTCTTGTGTGTTTTCTTCTCTATAACTACAATCTAGATTTTGTTCCATTCTATATGTCCATTAATTAGTATATTTACATGCTTGCATATAATTGAGGCCATTACTTGTTTTGCTCACTTATCCCAAATAAGCCTACCC**T/C**TTATGTCACCATTGTTAGCCACTTTGAGCCTTTTAATCCCCTCTGTTCTATAACCACATCACTAGCCTTAAGCAGAAAAATAATTAAATACCCCAATTGAATCTTTGGTTAACTTAAGATAGAGATTATGCATCAATTAAGTGTAGAGAAACTGTGGGAACATGGGTTAATAAAGGAATGTATCATGAATATTGG

GSS-99

>AdH1155-CPY2-O5TR

ATTAGGATGAGGAATAAAGATACATCTTAGAATAGAATCTAGCATAGTTTGAAATAGAATAGTGATATTATTAATCCATAAGAATCGGTAGAGCTCCTATCCTTAACCTAGGAGGTTAGTGACTCATAGCTTATAGAAATGGTAAAGTATTCGAATGGGGGAGGAGAAAGATCCTAAAATATGGGTGATCTTCTCCTATATATACTAATCTAATGAACAAGAATTACAGAAATAAGATTAACTAGCCTTGTAGTGCAAAAATTCACTTTTCAGGCACTCTTGGTGCGTGTTTGTGCTGAGCTTGAGTGACTGCACGAGTTGGTACTCCCTTATGGGCGTTGAACGCCAGCTTGGGACTCCTATTTGGGCTTTGGATGCCAGCTGCTCCCTTTTAAGCGTCCAACGCCAGGAAGAGGGTGAAGTGCTGGTGTTGAACGCCAGTTTTGGTCCTTCATTTCCTGAGAAAAGTATGCACTATTATATATTTCTAGAAAGCCCTGGATGTTAGCTTTCCATAGCCATTGAGAGCGCAGCATTCTGACTTCTATAGCTCCAGAAATGCTACTTCAAGTGCA**C/T**GGAGGTCAGATCCTGACAGCATCTGCACTCTTTTCTCTATCTCTGAATCATACTTTTTTCAAAGCTCCTCAATTTCAGCCAGAAAATATCTGAAATCATCATAAAATACACAAACTCAAAGTAGAATCCAAAAATATGAATTTATCACTAAAACCTATGAAAATATAATAAAACTTAAACAAAACATAATGAAAACTATATGAAAATGATGCCAAAAAGCGTAAAAACATCCGCTCATCAGCCAGCCAG
